# Psychological need satisfaction across work and personal life: an empirical test of a comprehensive typology

**DOI:** 10.3389/fpsyg.2023.1216450

**Published:** 2023-09-06

**Authors:** Claude Fernet, Alexandre J. S. Morin, Marcus B. Mueller, Nicolas Gillet, Stéphanie Austin

**Affiliations:** ^1^Département de gestion des ressources humaines, École de Gestion, Université du Québec à Trois-Rivières, Trois-Rivières, QC, Canada; ^2^Department of Psychology, Substantive-Methodological Synergy Research Laboratory, Concordia University, Montreal, QC, Canada; ^3^Department of Management, Jack Welch College of Business and Technology, Sacred Hearth University, Luxembourg, Luxembourg; ^4^Institut Universitaire de France, Paris, France; ^5^Département de psychologie, Université de Tours, Tours, France

**Keywords:** need satisfaction, work and personal life balance, latent profile analyses, well-being, job attitudes and behaviors

## Abstract

**Introduction:**

A comprehensive typology of the satisfaction of psychological needs at work and in personal life was developed and tested. The typology proposes five scenarios (*Enriched, Middling, Impoverished, Work-Fulfilled,* and *Personal Life-Fulfilled*) accounting for various profiles of employees showing distinct configurations of global and specific levels of need satisfaction at work and in personal life.

**Methods:**

The scenarios were tested in a sample of 1,024 employees.

**Results:**

Using latent profile analysis, five profiles were identified that were consistent with four or the five scenarios, either aligned (*Globally Satisfied, Globally Unsatisfied*) or misaligned (*Globally Satisfied at Work with High Relatedness, Globally Satisfied in Personal Life with High Autonomy, and Globally Satisfied in Personal Life with Low Autonomy*) across domains. No profile corresponding to the *Middling* scenario was observed.

**Discussion:**

The results indicate that perceived job and individual characteristics predicted membership in distinct profiles. More importantly, unlike the profile *Globally Unsatisfied*, the profile *Globally Satisfied* contributed substantially to higher well-being (vitality and lower psychological distress), and to more favorable job attitudes (job satisfaction and lower turnover intentions) and behaviors (self-rated job performance and lower absenteeism, presenteeism, and work injuries). Furthermore, two of the misaligned profiles were also substantially associated with highly desirable outcome levels.

## Introduction

A central tenet of Self-determination theory (SDT; Deci et al., 2017) is that satisfaction of three basic psychological needs—autonomy, competence, and relatedness—is required for optimal functioning and well-being. In the work context, Van den Broeck et al. (2016) provided meta-analytic support for this assumption, revealing that the satisfaction of these needs in employees is associated with key indicators of job attitudes (e.g., satisfaction, commitment), behaviors (e.g., performance, lower absenteeism), and well-being (e.g., engagement, lower burnout). Another central tenet of SDT is that optimal functioning requires that all three needs be fulfilled across important life domains, given that any contextual factors able to satisfy (or thwart) these needs are thought to lead to positive (or negative) effects on individuals’ general levels of well-being across domains (Deci and Ryan, 2008; Vansteenkiste et al., 2020). Nevertheless, although research provides valuable insights into the fundamental role of need satisfaction at work, it neglects employees’ need satisfaction in their personal life (i.e., the nonwork sphere of activity; Bulger et al., 2007), and how need satisfaction coexists at the interface of different domains. As noted by work-life interface scholars (e.g., Casper et al., 2018; Powell et al., 2019), the personal life has extended beyond the traditional family domain to encompass a wider diversity of spheres of activity (e.g., leisure, friends, social and community involvement) that must be reconciled.

The importance of considering the interface between work and personal life in relation to need satisfaction is evidenced by the fact that when an individual plays incompatible personal and professional roles, negative consequences are likely to spread to the work (e.g., job dissatisfaction, burnout) and personal life (e.g., life dissatisfaction, psychological distress) domains, which in turn may carry a toll for organizations (e.g., lower performance, absenteeism) (Kossek and Ozeki, 1998; Eby et al., 2005; Wayne et al., 2017). If optimal functioning and well-being across life spheres require the satisfaction of psychological needs across important life domains (Deci and Ryan, 2008), and if we acknowledge that the professional and personal domains need to operate in tandem (Wayne et al., 2017; Casper et al., 2018), it becomes necessary to determine how need satisfaction coexists at the interface of both domains to better understand the factors able to contribute to that satisfaction, along with potential consequences. However, little theorizing has focused on the specific configurations of need satisfaction at the work–personal life interface, and how these configurations may simultaneously predict job attitudes, behaviors, and well-being.

We address this issue by proposing that need satisfaction, at work and in personal life, matters for adaptive and healthy functioning across these two life domains. Building on Sheldon and Niemiec’s (2006) suggestion that the *balanced* satisfaction of all three psychological needs in a given domain may be even more important than the isolated satisfaction of each specific need, we also propose that balance might be equally important across domains. More precisely, we posit that alignment in the global amount of need satisfaction at work and in personal life as well as the degree of balance (or imbalance) in the satisfaction of each specific need in relation to this global level should help us better predict organizational and individual outcomes. By considering both aspects (alignment/misalignment across domains, and balance/imbalance among the three needs), we make three main contributions to research on need satisfaction. First, we propose a typology of possible scenarios of need satisfaction at the work–personal life interface to guide future research. This typology also considers the theoretical processes that underlie the diverse scenarios, and the expected consequences of these scenarios. Second, we also investigate the predictive role of job characteristics in relation to these scenarios to identify possible mechanisms under the control of organizations that may encourage the emergence of more adaptive work-life need satisfaction profiles among employees. Third, although the proposed typology aligns with SDT, it challenges the assumption that when there is a discrepancy or imbalance among the needs that are satisfied or unsatisfied within a specific life domain, then negative individual outcomes such as lower well-being are inevitably produced (Sheldon and Niemiec, 2006). By demonstrating the compensatory contribution of specific levels of need satisfaction when one domain (work or personal life) predominates in terms of global levels of need satisfaction, we contribute to identify the psychological mechanisms that may fuel job attitudes, work behaviors, and psychological well-being.

## Theory and hypotheses

### Self-determination theory and psychological need satisfaction

SDT (Deci and Ryan, 2000) posits that optimal functioning is anchored in the satisfaction of three psychological needs: Autonomy (the ability to initiate and regulate one’s behavior), competence (the capacity to take effective actions to achieve one’s goals), and relatedness (the feeling of having mutually satisfying relationships). Need satisfaction, and the ability of a specific life domain to nurture these needs, is expected to foster growth, development, and well-being (Deci and Ryan, 2000).

There is abundant support for this proposal in research focusing on the work domain (Van den Broeck et al., 2016), as well as in other life domains (e.g., education: Garn et al., 2019; sport: Gillet et al., 2009). Organizational studies show that need satisfaction at work is associated with favorable attitudes and behaviors as well as employee well-being in the same life domain (Van den Broeck et al., 2016). However, research is currently lacking on the combined role of need satisfaction occurring across the work and personal life domains. Furthermore, beyond this domain-specificity, the three basic needs are also generally assumed to be relatively independent from each other, and yet are expected to produce complementary effects (Ryan and Deci, 2017). However, research also shows that the degree of satisfaction of the three needs tends to be moderately to highly intercorrelated at work (Van den Broeck et al., 2016) and in other life domains (e.g., Garn et al., 2019; Gillet et al., 2020b), thus calling into question their effective independence.

### Balance and alignment in psychological need satisfaction

Sheldon and Niemiec (2006) proposed that, in addition to the amount of need satisfaction, a balance among the needs that are satisfied is essential for well-being. Thus, consistency, or balance, in the degree of satisfaction of the different needs (when satisfaction is similar across needs) in a given context should be more favorable to well-being than the imbalanced satisfaction of specific needs relative to others (when satisfaction differs across needs). Although theoretically appealing, empirical evidence is equivocal regarding the relations between balanced need satisfaction and indicators of functioning and well-being. The four pioneering studies by Sheldon and Niemiec (2006) showed that students who experienced balanced need satisfaction tended to report higher well-being, whereas a two-sample study by Dysvik et al. (2013) revealed weak correlations between balanced need satisfaction and intrinsic motivation in employees. In an additional attempt to investigate need satisfaction across multiple contexts, Milyavskaya et al. (2009) found that adolescents who reported *imbalanced* need satisfaction across contexts (at school, at home, with friends, and in job) had lower well-being. However, these authors (i.e., Milyavskaya et al., 2009; Dysvik et al., 2013) estimated the role of balanced need satisfaction by adding a difference scores to a model already including the main effects of each of the three needs and their interactions, thus introducing statistical redundancy (Gillet et al., 2019).

In light of these conflicting findings, Gillet et al. (2019, 2020a,b) challenged the indirect measurement of balanced need satisfaction (i.e., the degree of convergence in the satisfaction of the three needs) used in these studies, which involved the calculation of difference scores, known to be particularly sensitive to measurement errors (Edwards, 2002). They proposed bifactor modeling as the optimal way to obtain a direct, reliable, and meaningful indication of individuals’ global levels of need satisfaction across all three needs, together with an equally direct and non-redundant estimate of the degree of imbalance in the satisfaction of each specific need. In a bifactor model, domain-specific (work or personal life) ratings on all need satisfaction items are used to estimate one global factor (G-factor) reflecting participants’ global levels of need satisfaction in this domain. In addition to this global factor, all subscale-specific items are also used to define three orthogonal specific factors (S-factors) reflecting the variance uniquely associated with each need once the G-factor is taken into account (i.e., deviations from that global level). Gillet et al. (2019, 2020a,b) demonstrated that, because of this global-specific disagregation, the S-factors provide a direct representation of the degree of imbalance in the satisfaction of each need relative to all others in a specific domain.

Research evidence has since supported the value of a bifactor representation of need satisfaction across domains, including work (Sánchez-Oliva et al., 2017; Gillet et al., 2019, 2020a), education (Garn et al., 2019; Gillet et al., 2020b), sport (Brunet et al., 2016), and life in general (Tóth-Király et al., 2020). All of these studies have demonstrated associations between participants’ global levels of need satisfaction and a variety of indicators of psychological and behavioral functioning (e.g., burnout, anxiety, fatigue). Many of these studies also supported the additional role of imbalanced levels of satisfaction of specific needs in relation to the same outcomes. However, these studies are limited in their consideration of a single life domain.

### Toward a typology of psychological need satisfaction across work and personal life

Our main theoretical contribution lies in the development and validation of a typology designed to account for a range of possible need satisfaction scenarios at the work-personal life interface. These scenarios seek to account for different profiles of employees characterized by distinct amount (quantitative: More or less) and configuration (qualitative: More or less balanced) of global and specific need satisfaction at work and in personal life. This typology offers a holistic heuristic view of need satisfaction, and is derived from an fine-grained portrait that ventures beyond the impact of each need in the workplace (Gillet et al., 2019, 2020a,b) to also consider the personal life domain. Table 1 summarizes the main elements of the typology. In the next section, we elaborate on the theoretical underpinnings of five main scenarios in relation to functioning and well-being. When describing these scenarios, we use the term “alignment/misalignment” to refer to a situation where global levels of need satisfaction are similar across work and personal life domains, and the term “balance/imbalance” to refer to the situation where one specific need (i.e., competence, autonomy, and relatedness) is satisfied more (positive imbalance) or less (negative imbalance) than the other needs across contexts. However, rather than solely focusing on how balance/imbalance among all three needs within a specific life domain might impact functioning, we primarily focus on their alignment/misalignment across domains. Given the relative lack of previous research likely to offer guidance to this study, we felt that it was more critical to discuss theoretical scenarios with an emphasis on how global levels of need satisfaction were likely to display alignment or misalignment across domains, and that it would be premature to attempt to further differentiate these scenarios based on balanced/imbalanced levels of need satisfaction. However, we highlight how balance/imbalance might be expressed within each scenario.

**Table 1:** A typology of need satisfaction scenarios across work and personal life.

Theoretical scenario	Theoretical implications	Regulatory styles	Corresponding processes	Theoretical outcomes
**1. Aligned domains**	**Aligned global need satisfaction**			
1a. Enriched	Globally satisfied at work and in personal life, with (im)balanced specific need satisfaction	Autonomous, integrated self-regulated actions	Full internalization	Full functioning
1b. Middling	Globally, by moderately, satisfied at work and in personal life with (im)balanced specific need satisfaction	Autonomous, identified self-regulated actions	Almost full internalization	Moderate-to-high functioning
1c. Impoverished	Globally unsatisfied at work and in personal life with (im)balanced specific need satisfaction	Amotivated actions	No internalization, unintentionality, resignation	Poor functioning
**2. Misaligned domains**	**Misaligned global need satisfaction**			
2a. Work-fulfilled	Predominant global satisfaction at work, with (im)balanced specific need satisfaction	Autonomous combined with controlled actions	Accommodation, compensatory modes if unfulfilled needs	High domain- functioning, with some impairment
2b. Personal life-fulfilled	Predominant global satisfaction in personal life, with (im)balanced specific need satisfaction	Autonomous combined with controlled actions	Accommodation, compensatory modes if unfulfilled needs	High domain- functioning, with some impairment

### When global need satisfaction shows alignment across domains

The first three scenarios presuppose that global levels of needs at work and in personal life are similarly satisfied. These scenarios should result in employees’ profiles showing an alignment between global need satisfaction at work and in personal life, consistent with the organismic view underpinning SDT, which assumes that individuals naturally develop in the direction of increasing adaptation, integration, and coherence when possible (Vansteenkiste et al., 2020). These scenarios also relate to the notion of resources caravans from the conservation of resources (COR) theory (Hobfoll, 2002), which states that psychological resources lead to the accumulation of further resources. Because key psychological resources such as global need satisfaction tend not to exist in isolation, their impacts are likely to hold across life contexts.

The first scenario, called ***Enriched***, describes individuals characterized by high global levels of need satisfaction at work and in personal life. These individuals thus operate in environments that meet their psychological needs. Their actions are thought to be mainly autonomously regulated, meaning that they act out of choice and interest, leading to internalization (Ryan and Deci, 2017). This is because behaviors associated with basic need satisfaction tend to provide adaptive advantages when they are congruent with personal goals and values (Ryan and Deci, 2017). At work and in their personal life, these individuals should manifest well-being, attitudes and behaviors that are fully functional as long as they experience low imbalance in the satisfaction of their specific needs. Although we can reasonably expect these individuals to present lower imbalance of specific needs (Gillet et al., 2019) because of their high global levels, this scenario does not exclude the possibility that some may exhibit a more, presumably negative, imbalanced configuration when their specific needs are fulfilled to a slightly lower extent than their otherwise high global levels of need satisfaction.

A second scenario, called ***Middling***, describes individuals whose global levels of need satisfaction reflects neither an enriched, nor an impoverished, scenario, but is characterized by average need satisfaction across domains. These individuals evolve in environments in which need satisfaction is not an issue, but which are also not overly stimulating. At work, their basic needs are routinely met, but they tend not to get over-involved or withdrawn. Likewise, their personal life generally satisfies their basic needs, but without being particularly demanding or challenging. These individuals should function fairly well in both domains, as they engage in actions that are considered important but not necessarily integrated with their larger system of goals and values. They may display an active involvement across domains, but without being highly engaged or exhausted. These individuals should display no apparent signs of distress or of extreme wellness, and their attitudes should be consistently moderate (e.g., they should present reasonable levels of satisfaction and commitment to their job and organization), as would their behaviors (e.g., they should display adequate levels of job performance, without demonstrating many discretionary behaviors). Due to the nature of this scenario, positive and negative forms of imbalance in the satisfaction of specific needs remain possible, but not likely.

The third scenario, called ***Impoverished***, describes individuals with low global need satisfaction at work and in personal life. They occupy environments that fail to satisfy their psychological needs. Their actions are thought to be only weakly motivated as they have no real will to act for intrinsic or extrinsic reasons (Ryan and Deci, 2017). According to the organismic view and the COR loss spiral (i.e., any loss of resources tends to generate further losses), the outcomes would be lower well-being and functioning at work and in personal life. These individuals should present more symptoms of ill-being (e.g., job burnout) or alienation (e.g., psychological distress, somatization, lack of energy and vitality), especially if they present higher levels of imbalance in the satisfaction of their specific needs. Due to the low global levels of need satisfaction across domains observed in this scenario, an imbalanced configuration is likely (Gillet et al., 2020a), and should be primarily positive (i.e., some specific needs are met to a greater extent than the otherwise low global levels of need satisfaction). Gillet et al. (2019, 2020a) showed that this type of imbalance at work could be harmful for both attitudes (e.g., job satisfaction) and well-being (e.g., negative affect, burnout, fatigue).

### When global need satisfaction shows misalignment across domains

The next two scenarios theorize a misalignment between need satisfaction favoring work over personal life, or personal life over work. Such misalignment does not mean that need satisfaction is not important in a given context, simply that satisfaction is predominant (i.e., higher) in one domain. Despite the inherent tendency of individuals to seek consistency, some environments are inevitably more inclined to facilitate need satisfaction, while others are more likely to fail to meet individuals’ basic requirements (Deci and Ryan, 2000). When this happens, we propose that the degree of balance, positive or negative imbalance in the satisfaction of each specific need in relation to this global level should be particularly important in predicting key organizational and individual outcomes.

The fourth scenario, called ***Work-Fulfilled***, describes individuals with high global levels of need satisfaction at work, but lower global need satisfaction in their personal life. This scenario could correspond to a variety of profiles depending on the configuration of the specific satisfied needs. Thus, despite the global satisfaction of work-related needs, some individuals could still present imbalanced (positive or negative) levels of satisfaction of their specific needs. For instance, some employees might be exposed to particularly fulfilling work lives despite having less than satisfactory personal lives. Yet, these individuals might also experience a higher level of satisfaction of their specific need for relatedness due to the presence of highly supportive individuals in their personal network (a form of positive imbalance, likely to yield positive effects). In contrast, these same individuals might also experience a lower specific level of autonomy need satisfaction (a negative form of imbalance likely to yield negative effects) due to exposure to an overly controlling spousal partner or over-involvement in their community. When the environment only allows for the partial satisfaction of global needs at work (or in personal life in the next scenario), especially when coupled with a negative imbalance, individual actions would be driven simultaneously by autonomous regulations (pleasure, interest, or values) and controlled regulations (internal or external pressures), resulting in an incompletely integrated internalization process (Deci and Ryan, 2000). The use of accommodation mechanisms, such as compensatory motives (e.g., overinvestment in a job where one feels competent or appreciated), would allow for some collateral satisfaction with a life that generates imbalance in the satisfaction of specific needs (Deci, 1980). Global need satisfaction at work would certainly offer these individuals the psychological nutrients they need to be highly functional at work, although a negative imbalance may still lead them to experience some impairment in functioning and well-being.

Inversely, the fifth scenario, called ***Personal Life-Fulfilled***, describes individuals with higher global levels of need satisfaction in their personal life relative to their work life. This scenario could also include diverse profiles, depending on the configuration of the specific needs that are satisfied (positive vs. negative imbalance). For instance, some might be able to benefit from a fulfilling personal life, despite exposure to a highly stressful and demanding work life. Yet, these individuals might also experience particularly low specific levels of competence need satisfaction due to an overly controlling work supervisor (a negative form of imbalance), or be able to benefit from higher specific levels of relatedness need satisfaction due to particularly appreciated colleagues. These individuals would show similar types of behavioral regulations (both autonomous and controlled) as those from the previous scenario, and be fully functional in their personal life, although their well-being could be impaired, especially if they present a negative imbalance of specific needs.

### A person-centered approach to the investigation of work-life need satisfaction scenarios

Investigation of these scenarios requires the adoption of a person-centered approach, which is specifically designed to identify subpopulations, or profiles, of individuals displaying a different configuration on a set of indicators (Meyer and Morin, 2016). Statistical research has demonstrated that whenever profile indicators display a dual global/specific nature, such as ratings of need satisfaction, this dual nature needs to be taken into account (Morin et al., 2016, 2017). Indeed, failure to do so results in the erroneous identification of profiles differing only quantitatively (i.e., sharing the same high, moderate, or low level across indicators), thus hiding meaningful qualitative differences that appear only when considering the dual global/specific nature of the indicators. When anchored in a bifactor representation of need satisfaction, scores on the latent factors reflecting each specific need are directly expressed in terms of balance (corresponding to a score of 0 on the latent factor reflecting these needs) or imbalance (corresponding by scores higher or lower than 0).

We are aware of two studies that have sought to identify need satisfaction profiles at work. Unfortunately, Esdar et al. (2016) failed to properly disaggregate employees’ global and specific levels of need satisfaction, leading to the identification of two profiles differing only quantitatively from one another (displaying high or low levels across all three needs), and of two other profiles dominated, respectively, by higher levels of competence or autonomy need satisfaction. In contrast, Gillet et al. (2019) were able to identify four profiles while relying on a bifactor disaggregation of employees global and specific (i.e., imbalance) levels of need satisfaction. The first of those profiles (*Normative*) was a large (i.e., 77.13%), characterized by average and balanced levels of need satisfaction across indicators. Although this study only considered one side of the coin (the work domain), this profile seems to match the *Middling* scenario. In contrast, the other profiles displayed low to very low global levels of need satisfaction, and a strong imbalance across all three needs. Due to their positive imbalance, these profiles may correspond either to the *Impoverished* scenario, or to the flip side of the coin of the *Personal Life-Fulfilled* scenario. These profiles were also replicated across two samples of employees and found to share well-differentiated relations with a series of predictors related to job demands and resources, and outcomes related to anxiety and physical fatigue.

Adopting a similar methodology, Gillet et al. (2020b) identified five profiles in the educational area, which were also generally consistent with our scenarios. Four of their profiles were consistent with the idea that imbalanced levels of need satisfaction were more frequent among profiles characterized by lower global levels of need satisfaction, whereas the last one suggested that even highly satisfied individuals could display imbalanced levels of need satisfaction, although in this case this imbalance was harmful. On the basis of these theoretical (i.e., the typology), methodological (i.e., the need to disaggregate global and specific levels of need satisfaction), and empirical (i.e., previous research results) considerations, we propose a first general hypothesis:

Hypothesis 1: The observed profiles will reflect the theoretical scenarios: *Enriched, Middling, Impoverished, Work-Fulfilled*, and *Personal Life-Fulfilled*.

### Predictors of need satisfaction profiles across work and personal life

SDT proposes that need satisfaction varies across individuals according to their life contexts (Deci and Ryan, 2000). In this regard, accumulating evidence indicates that job characteristics—insofar as they support or hinder psychological need satisfaction—have considerable influence on work attitudes (job satisfaction, commitment, and turnover intentions) and behaviors (self-rated job performance), but also over broader manifestations of well-being that spill over into personal life, such as psychological distress and life satisfaction (Van den Broeck et al., 2016). Inspired by the job demands–resources (JD-R) model (Schaufeli and Bakker, 2004), some studies suggest that, in contrast to job demands (e.g., role overload and ambiguity, emotional demands), job resources (e.g., social support, job control, recognition, fairness) foster functioning and well-being at work through need satisfaction (e.g., Gillet et al., 2012; Fernet et al., 2013; Trépanier et al., 2015a; Huyghebaert et al., 2018).

Gillet et al. (2019, 2020a) showed that job demands (e.g., role ambiguity, mental load) and resources (e.g., decisional participation, task autonomy and identity, colleague and organizational support) that distinctly influenced on global and specific levels of need satisfaction also predicted (depending on the balance) diverse indicators of job attitudes and behaviors (e.g., helping behaviors, sportsmanship) and well-being (e.g., positive and negative affect, cognitive weariness). Although these predictors relate mainly to work, they are likely to cross over from the psychological experience at work to impact need satisfaction in personal life. This rationale is based on the scarcity hypothesis (Chapman et al., 1994), which posits that individuals have a finite amount of time and energy, so that work and personal life compete for the same resources. Thus, overworked employees might increase their efforts in the evenings and on weekends to catch up, which would prevent them from achieving other highly valued goals. Likewise, individuals who struggle in a job environment that provides insufficient resources might believe that, despite their best efforts, their needs will never be adequately met. Indeed, there is evidence that limited opportunities to exert control at work results in a more passive or sedentary lifestyle after work (e.g., Brisson et al., 2000), involving social isolation (Bourbonnais et al., 2001), less leisure activities (Karasek and Theorell, 1990), and lower physical activity (Hellerstedt and Jeffery, 1997; Hervieux et al., 2022). Besides being consistent with SDT (Deci et al., 2017), this reasoning also aligns with several resource-adaptation models assuming that functioning and well-being depend on the possibility and capability to actively engage with the environment (Karasek and Theorell, 1990; Hobfoll, 2002).

We recognize that personal life demands and resources are also likely to be involved in need satisfaction across the work and personal life domains. However, as a first attempt to consider need satisfaction at the interface of these domains, we take a first step to consider the role played by work characteristics, in order to provide some guidance to organizations willing to invest into improving their workers work-life need satisfaction. We focus on four job characteristics: *Workload* (amount of work required of an employee; Spector and Jex, 1998), *job control* (opportunities to make decisions and exercise control over the tasks to be accomplished; Karasek and Theorell, 1990), *fairness* (perceptions that decisions and resource allocation at work are fair and equitable and that people are treated with consideration and respect; Cropanzano and Greenberg, 1997), and sense of *community* (the quality of social interactions at work, including conflict, mutual support, closeness, and the capacity to work as a team; Leiter and Maslach, 2000). Thus, the workplace should help satisfy employees’ psychological needs when they perceive that it provides them with reasonable workloads and adequate resources (Fernet et al., 2013; Trépanier et al., 2015a; Gillet et al., 2019, 2020a). Furthermore, need satisfaction at work should also influence personal life. This reasoning is consistent with the work-home resources (W-HR) model (Ten Brummelhuis and Bakker, 2012), which proposes a loss spiral process whereby expanding personal resources to cope with job demands or having insufficient job resources can induce further losses due to the need to face personal life demands with resources already expanded at work. Given the limited nature of individual resources (Hobfoll, 2002), employees facing high job demands or low job resources may also come to see work as preventing them from fully satisfying their psychological needs at work and in their personal life. We thus propose that:

Hypothesis 2a: Perceptions of high workload increase the likelihood of membership in profiles characterized by lower global levels of need satisfaction at work and in personal life (the *Impoverished* scenario) or by higher global levels of need satisfaction predominantly at work or in personal life combined with low levels of need satisfaction across specific needs (the *Work- and Personal Life-Fulfilled* scenarios).

Hypothesis 2a: Positive perceptions of job resources (fairness, control, and sense of community) increase the likelihood of membership in profiles characterized by higher global levels of need satisfaction at work and in personal life (the *Enriched* scenario, followed by the *Middling* scenario) or by higher global levels of need satisfaction predominantly at work or in personal life combined with high levels of need satisfaction across specific needs (the *Work- and Personal Life-Fulfilled* scenarios).

Beyond job characteristics, another relevant question is whether variables such as individual characteristics (age, sex, organizational tenure, relationship status, and parental status) add to the prediction of need satisfaction profiles at work and in personal life (Tóth-Király et al., 2020). Although these variables appear to play a negligible role in need satisfaction at work (Van den Broeck et al., 2016), they could make a more substantial contribution to need satisfaction in personal life (presumably in the presence of misalignment across domains), and to specific configurations of need satisfaction across domains. On this point, some studies suggest that a variety of individual outcomes closely linked to need satisfaction vary according to certain demographic characteristics, over and above job and personal life stressors (Marchand et al., 2015; Bilodeau et al., 2020). For example, lower psychological distress was observed among older male employees (Marchand and Blanc, 2010), women facing family to work conflict (Bilodeau et al., 2020) and employees who lived as a couple or with dependent children (Marchand et al., 2015). Although these studies tie these individual characteristics to overall well-being, research has yet to test these relations with respect to need satisfaction across work and personal life domains (known to represent a core driver of well-being). Testing these associations may thus offer additional insights into the mechanisms involved in the associations between need satisfaction profiles and well-being. However, due to the small number of studies able to provide guidelines, it is difficult to formulate clear expectations for the contribution of demographic variables to need satisfaction profiles at work and in personal life. Accordingly, we leave as an open research question whether the above-mentioned individual characteristics relate to the likelihood of membership in the various need satisfaction profiles.

### Organizational and individual outcomes of need satisfaction profiles

To confront our scenarios with data and ensure that they have real empirical value, we contrasted them on a series of organizational and individual outcomes. Because few studies, if any, have considered psychological need satisfaction simultaneously at work and in personal life, we chose to use well-documented organizational (attitudinal and behavioral) and individual (overall well-being) outcomes of need satisfaction at work (Van den Broeck et al., 2016) that are also liable to be associated with need satisfaction in personal life. More specifically, the attitudinal outcomes are *job satisfaction* (i.e., overall job satisfaction; Fouquereau and Rioux, 2002) and *turnover intentions* (i.e., a conscious and deliberate willingness to leave the organization; Tett and Meyer, 1993) and the behavioral outcomes are *self-rated job performance* (i.e., the performance quality of core job activities; Abramis, 1994), *sickness absenteeism* (i.e., number of sick days and *absence occurrence*; Kessler et al., 2003), *sickness presenteeism* (i.e., attending work while ill; Robertson and Cooper, 2011), and work *injury absenteeism* (i.e., number of days missed due to work accidents and work injury incidents). In the absence of specific personal life indicators (e.g., life satisfaction, couple satisfaction, community involvement), we focus on two global (not domain-specific) well-being outcomes assumed to be impacted by the work-life interface: *psychological distress* (i.e., nonspecific symptoms of impaired psychological health; Kessler et al., 2002) and *subjective vitality* (i.e., positive feelings of aliveness and energy; Ryan and Frederick, 1997). These decontextualized indicators of well-being are sensitive to variations in job and personal life characteristics (Marchand et al., 2015). For instance, in an eight-year prospective study, Marchand and Blanc (2010) found that, compared to job factors, the effects of non-job factors (e.g., marital status, couple- and child-related strain) and individual factors (e.g., age, sex) were substantially higher on the onset of psychological distress. We thus propose that:

Hypothesis 3: Profiles characterized by higher global levels of need satisfaction at work and in personal life (the *Enriched* scenario, followed by the *Middling* scenario) or by higher global levels of need satisfaction predominantly at work or in personal life combined with high levels of need satisfaction across specific needs (the *Work- and Personal Life-Fulfilled* scenarios), are positively associated with more favorable organizational outcomes (job attitudes: Higher satisfaction and lower turnover intentions; and job behaviors: Higher self-rated job performance and lower absenteeism, presenteeism, and injuries; H3A) and individual outcomes (overall well-being: Higher vitality and lower psychological distress; H3B).

## Method

### Data and sample

Data were collected among a sample of employees working for an European transport company. Potential participants were contacted via an email explaining the study’s purpose, emphasizing its anonymous and voluntary nature, and including a link to an online questionnaire. No compensation was provided. The final sample includes 1,024 participants (33.3% women) with an average age of 40.04 years (SD = 8.48) and 12.47 years (SD = 8.24) of tenure in the organization. Most held a permanent position (86%), were living as a couple (82%), and had at least one dependent child (58%). Regarding education, 43.4% had secondary or vocational education, 31.2% had post-secondary education, and 25.4% had university education.

### Measures

All measures were administered in English (*N* = 90), German (*N* = 370), or French (*N* = 564). Measures not previously validated in German and French were adapted using a classical translation back-translation procedure (Brislin, 1980) involving independent translators.

#### Need satisfaction

We used an adapted version (Sheldon et al., 2001) of the Basic Need Satisfaction Scale (Deci et al., 2001) to assess the extent to which participants experienced satisfaction of their basic needs at work and in personal life (9 items per domain). Sample items are, “I am free to express my ideas and opinions [on the job (or) at work]” [in my personal life] (autonomy; α =0.69 at work, α = 0.61 in personal life); “Most days I feel a sense of accomplishment [at my job(or)in my work]” [in my personal life] (competence; α =0.45 at work, α = 46 in personal life); and “People [at work] care about me” [in my personal life] (relatedness; α =0.57 at work, α = 0.57 in personal life) ^1^. Items were scored on a seven-point scale ranging from 1 (Not at all true) to 7 (Very true).

#### Job characteristics

Four job characteristics were assessed with subscales from the Areas of Work Life Scale (AWS; Leiter and Maslach, 2004): *Work overload* (5 items, α =0.66; e.g., “I do not have time to do the work that must be done”), *job control* (4 items, α =0.70, e.g., “I have control over how I do my work”), *fairness* (6 items, α =0.80, e.g., “Management treats all employees fairly”), and sense of *community* (5 items, α =0.75, e.g., “People trust one another to fulfill their roles”). All items were scored on a scale from 1 (Strongly disagree) to 5 (Strongly agree).

#### Job attitudes

*Job satisfaction* was assessed with five items (α = 0.86; e.g., “I am satisfied with my job”) from the Satisfaction with Life Scale (Diener et al., 1985) while replacing the word “life” with “job” (Gillet et al., 2012). A sample item is, “I am satisfied with my job.” Items were rated on a seven-point scale ranging from 1 (Strongly disagree) to 7 (Strongly agree). Fouquereau and Rioux (2002) demonstrated the scale’s score reliability and construct validity. *Turnover intentions* were assessed with three items adapted from O’Driscoll and Beehr (1994), e.g., “I have thought about leaving my job” (α = 0.70). Items were scored on a seven-point scale ranging from 1 (Strongly disagree) to 7 (Strongly agree).

#### Job behaviors

*Self-rated job performance* was assessed with a single item (“How often do you feel you can do your best quality work at your job”) rated on a five-point scale from 1 (Never) to 5 (Always). *Sickness presenteeism* was measured using a single item (“Over the last 12 months, for how many days did you come in to work even though you were ill or injured?”). Similarly, *sickness absenteeism* was measured with a single item (“Over the last 3 months, how many days of work did you have to miss due to illness or injury?). Single self-report items are commonly used to assess attendance (Skagen and Collins, 2016). However, we complemented this data with the organization’s official reports of employee absenteeism (*number of days of absence* and *absence occurrence*). Absence occurrence refers to absence from work regardless of the length in days. In addition, we considered employee work injuries (*number of days missed due to a work accident* and *work injury incidents*).

#### General well-being

*Psychological distress* was assessed with the K6 (Kessler et al., 2002). This six-item instrument (α = 0.86) measures non-specific symptoms of anxiety and depression experienced during the previous month (feeling nervous, hopeless, restless or fidgety, so sad that nothing could cheer you up, that everything was an effort, and worthless). Items were rated on a five-point scale ranging from 1 (Never) to 5 (Always). *Subjective vitality* was assessed with six items (Bostic et al., 2000) adapted from Ryan and Frederick (1997) to measure feelings of aliveness and energy. A sample item is, “I feel alive and vital” (α = 0.89). All items were rated on a seven-point scale ranging from 1 (Never true) to 7 (Always true).

### Analysis

#### Preliminary measurement models

We first contrasted preliminary measurement models to identify the optimal representation of need satisfaction ratings across work and personal life. Supporting mounting evidence suggesting that a bifactor representation is most suitable for need satisfaction ratings across life domains (e.g., Garn et al., 2019; Gillet et al., 2019, 2020b), these analyses led us to select a bifactor solution including two correlated domain-specific G-factors (work satisfaction and personal life satisfaction) and three domain-general S-factors (autonomy, competence, and relatedness) reflecting deviations from the G-factors. Both G-factors were well-defined (ω = 0.804 at work and 0.815 in personal life) and relatively independent (*r* = 0.159), supporting the domain-specificity of global need satisfaction ratings.

Although the S-factors were weaker (ω = 0.486 for autonomy, 0.432 for relatedness, and 0.222 for competence), both the autonomy and relatedness S-factors retained meaningful, albeit low, specificity anchored in a subset of items presenting construct-relevant variance left unexplained by the G-factors. Because bifactor models separate reliable variance in two sets of factors, leniency has been advocated in the interpretation of omega values taken from bifactor models, suggesting that values approaching 0.500 should be considered acceptable (e.g., Perreira et al., 2018; Morin et al., 2020). Conversely, the competence S-factor appears to “vanish” once the variance explained by the G-factors is considered, suggesting that these items only retain a limited amount of specificity once the variance explained by the global factor(s) is considered (Morin et al., 2020). Observing such vanishing S-factors is the norm in studies relying on a bifactor approach to need satisfaction measurement (Gillet et al., 2019, 2020a, 2020b). Our results thus simply suggest that competence need satisfaction only retains negligible amounts of imbalance relative to global levels of need satisfaction. Given that the main effect of unreliability is to reduce the size of factor correlations, it is unlikely to play a role in a study relying on indicators estimated from orthogonal (uncorrelated) factor scores taken from a bifactor model to estimate latent profile analyses (estimated from the multivariate distribution of these factor scores rather than their covariances; McLachlan and Peel, 2000). In this regard the incorporation of unreliable indicators would simply result in this indicator not being relevant to the differentiation between the profile (i.e., being close to average). However, it is also possible for an empty S-factor to retain a little specificity limited to only a subset of participants corresponding to one or two profiles, in which case this S-factor may emerge as a defining characteristic of these profiles.

Details on these preliminary analyses (including a more complete discussion of the vanishing S-factor) are reported in the first (need satisfaction; including Supplementary Tables S1–S3) and second (predictors and outcomes; including Supplementary Tables S4, S5) of the online supplements. Correlations are reported in Supplementary Table S6.

#### Latent profile analyses

All analyses were conducted using Mplus 8.3 (Muthén and Muthén, 2019) maximum likelihood robust (MLR) estimator. Due to our online setup, there was no missing data. The need satisfaction, predictors, and outcome indicators were factor scores (estimated in standardized units with *M* = 0 and SD = 1) saved from our preliminary analyses. Factor scores have the ability to achieve a partial correction for unreliability (Skrondal and Laake, 2001) and to preserve the nature of the measurement model (e.g., bifactor models; Morin et al., 2016, 2017).

LPA solutions including one to eight need satisfaction profiles were first estimated. In these solutions, the means and variances of the indicators were estimated freely across profiles (Peugh and Fan, 2013). These solutions were estimated with 10,000 random starts, 1,000 iterations, and 500 optimizations (Hipp and Bauer, 2006). To identify the optimal number of profiles, various sources of information need to be considered, including the statistical adequacy of the solution and the theoretical and heuristic value of the profiles (Muthén, 2003; Morin and Litalien, 2019). Statistical indices can support this decision: The Bayesian Information Criterion (BIC) and its sample-size adjusted version (ABIC), the Akaïke Information Criterion (AIC) and its consistent version (CAIC), the adjusted Lo et al.’s (2001) Likelihood Ratio Test (aLMR), and the Bootstrap Likelihood Ratio Test (BLRT). Lower AIC, CAIC, BIC, and ABIC values suggest a better-fitting model. A statistically significant aLMR and BLRT indicate that a model may be better than one including fewer profiles.

Statistical research supports the efficacy of four of these indicators (CAIC, BIC, ABIC, and BLRT), but not that of the AIC and aLMR (e.g., Nylund et al., 2007; Peugh and Fan, 2013; Tein et al., 2013; Diallo et al., 2016, 2017). Although we report the AIC and aLMR for purposes of full disclosure, these indicators will not be used. Moreover, all of these indicators sometimes keep on suggesting the addition of profiles (Marsh et al., 2009). When this happens, one should look at the point at which they reach a plateau and contrast the solutions around this plateau using theory and logic (e.g., Morin et al., 2011a,b; Morin and Litalien, 2019). We finally report the entropy, as a useful summary of the classification accuracy of participants into profiles (ranging from 0 to 1).

Relations between the predictors and profile membership were assessed with a multinomial logistic regression link function following the direct inclusion of predictors (Diallo et al., 2017). Outcome levels were also contrasted across profiles using a model-based procedure developed by Lanza et al. (2013) implemented using Auxiliary (DCON) (Asparouhov and Muthén, 2014).

## Results

### LPA

**Figure 1:**
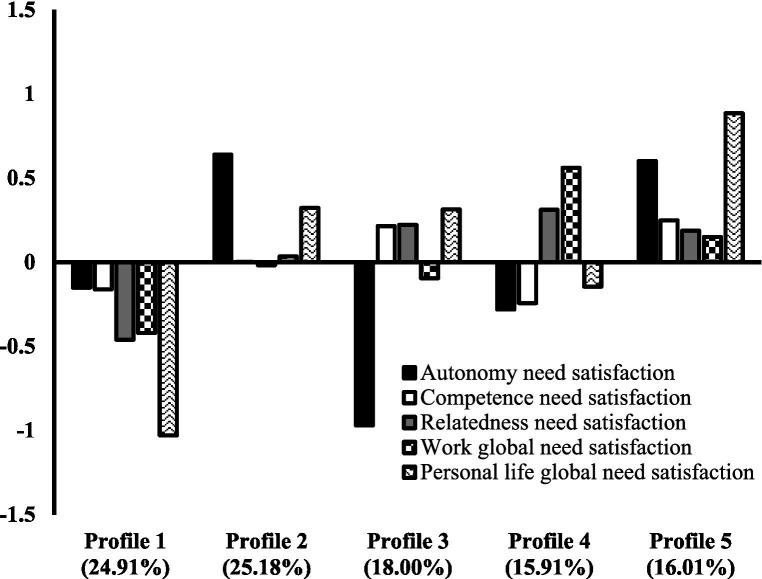
Final five-profile solution. Profile 1: *Globally Unsatisfied*; Profile 2: *Globally Satisfied in Personal Life with High Autonomy*; Profile 3: *Globally Satisfied in Personal Life with Low Autonomy*; Profile 4: *Globally Satisfied at Work with High Relatedness*; and Profile 5: *Globally Satisfied*; Profile indicators are factor scores estimated in standardized units with a mean of 0 and a standard deviation of 1.

The statistical indicators associated with the alternative LPA solutions are reported in Table 2. Whereas the CAIC reached its lowest point at six profiles, the BIC reaches it at seven profiles. Neither the BLRT nor the ABIC converged on any specific solution. The decreases in CAIC, BIC, and ABIC reached a plateau closer to three or four profiles. Solutions including three to six profiles were thus inspected more carefully. This examination revealed that up to five profiles, new profiles were reasonably large and had a meaningful contribution (e.g., considering the final solution reported in Figure 1, only Profiles 1 and 5 were already present in the three-profile solution). In contrast, adding profiles beyond this solution only resulted in the arbitrary division of an existing profile into much smaller ones (approximately 1% of the sample). For this reason, the five-profile solution was retained for interpretation.^2^ This solution is associated with a reasonably good level of classification accuracy (ranging from 63.9 to 91.2% across profiles, as shown in Supplementary Table S7 of the online supplements). This final model is illustrated in Figure 1 and results are reported in Supplementary Table S8 of the online supplements.

**Table 2:** Results from the latent profile analysis models.

Model	LL	#fp	Scaling	AIC	CAIC	BIC	ABIC	Entropy	aLMR	BLRT
1 Profile	−6090.691	10	1.195	12201.383	12260.698	12250.698	12218.937	Na	Na	Na
2 Profiles	−5703.381	21	1.104	11448.762	11573.322	11552.322	11485.624	0.638	<0.001	<0.001
3 Profiles	−5535.254	32	1.056	11134.507	11324.315	11292.315	11190.679	0.708	<0.001	<0.001
4 Profiles	−5480.694	43	1.143	11047.388	11302.441	11259.441	11122.868	0.691	0.063	<0.001
5 Profiles	−5430.721	54	1.108	10969.422	11289.741	11235.741	11064.232	0.676	0.072	<0.001
6 Profiles	−5382.489	65	1.065	10894.978	11280.524	11215.524	11009.077	0.709	0.063	<0.001
7 Profiles	−5343.994	76	1.339	10839.988	11290.780	11214.780	10973.396	0.695	0.777	<0.001
8 Profiles	−5312.927	87	1.140	10799.854	11315.892	11228.892	10952.571	0.687	0.330	<0.001

LL, Model loglikelihood; #fp, Number of free parameters; scaling, Scaling correction factor associated with robust maximum likelihood estimates; AIC, Akaïke information criteria; CAIC, Constant AIC; BIC, Bayesian information criteria; ABIC, Sample-size adjusted BIC; aLMR, Adjusted Lo–Mendel–Rubin likelihood ratio test; BLRT, Bootstrap likelihood ratio test.

Profile 1 was characterized by low levels of global need satisfaction at work and very low levels of global need satisfaction in personal life, with moderately low to low specific levels of domain-general autonomy, competence, and relatedness need satisfaction. This *Globally Unsatisfied* profile corresponds to 24.91% of the sample. Profile 2 was characterized by average levels of global need satisfaction at work and high levels of global need satisfaction in personal life domain, with very high specific levels of domain-general autonomy need satisfaction and average specific levels of domain-general competence and relatedness need satisfaction. This *Globally Satisfied in Personal Life with High Autonomy* profile corresponds to 25.18% of the sample. Profile 3 was characterized by moderately low levels of global need satisfaction at work and high levels of global need satisfaction in personal life, with moderately high specific levels of domain-general competence and relatedness need satisfaction and very low specific levels of domain-general autonomy need satisfaction. This *Globally Satisfied in Personal Life with Low Autonomy* profile corresponded to 18.00% of the sample. Profile 4 was characterized by high levels of global need satisfaction at work and moderately low levels of global need satisfaction in personal life, with moderately low specific levels of domain-general autonomy and competence need satisfaction and high specific levels of domain-general relatedness need satisfaction. This *Globally Satisfied at Work with High Relatedness* profile corresponded to 15.91% of the sample. Finally, Profile 5 was characterized by moderately high levels of global need satisfaction at work and very high levels of global need satisfaction in personal life, with moderately high to high specific levels of domain-general autonomy, competence, and relatedness need satisfaction. This *Globally Satisfied* profile characterized 16.01% of the participants. These profiles tend to support Hypothesis 1, displaying consistence with four of the five theoretical scenarios proposed in our typology, with two profiles matching the *Personal Life-Fulfilled* scenario. No profile corresponding to the *Middling* scenario was observed.

### Need satisfaction profiles: predictors

The results from the multinomial regression analyses assessing the relations between predictors and the likelihood of profile membership are reported in Table 3.

**Table 3:** Results from multinomial logistic regressions for the effects of the predictors and demographic variables on profile membership.

	Profile 1 vs. Profile 5		Profile 2 vs. Profile 5		Profile 3 vs. Profile 5		Profile 4 vs. Profile 5		Profile 1 vs. Profile 4	
	Coef. (SE)	OR	Coef. (SE)	OR	Coef. (SE)	OR	Coef. (SE)	OR	Coef. (SE)	OR
Sex (women)	−1.686 (0.313)**	0.185	−1.145 (0.299)**	0.318	−0.389 (0.303)	0.678	−0.491 (0.312)	0.612	−1.195 (0.369)**	0.303
Age	0.013 (0.142)	1.013	−0.262 (0.157)	0.769	−0.455 (0.160)**	0.634	−0.301 (0.164)	0.740	0.313 (0.175)	1.368
Org. tenure	−0.216 (0.131)	0.806	−0.134 (0.150)	0.875	0.052 (0.151)	1.053	0.173 (0.153)	1.188	−0.389 (0.159)*	0.678
Education	−0.024 (0.131)	0.977	0.145 (0.153)	1.156	0.378 (0.144)**	1.460	0.519 (0.158)**	1.680	−0.542 (0.154)**	0.582
Rel. Status	−1.018 (0.379)**	0.361	−0.529 (0.394)	0.589	−0.114 (0.458)	0.892	−0.663 (0.414)	0.515	−0.355 (0.436)	0.701
#Children	0.392 (0.127)**	1.480	0.172 (0.148)	1.188	0.319 (0.145)*	1.376	0.206 (0.154)	1.228	0.186 (0.155)	1.205
Workload	0.363 (0.171)*	1.438	−0.084 (0.194)	0.920	0.514 (0.187)**	1.673	0.289 (0.193)	1.335	0.074 (0.192)	1.077
Control	0.133 (0.234)	1.143	−0.074 (0.257)	0.929	−0.250 (0.248)	0.779	0.343 (0.157)*	1.409	−0.210 (0.230)	0.811
Community	−0.852 (0.225)**	0.427	−0.453 (0.242)	0.636	−0.172 (0.242)	0.842	0.331 (0.135)*	1.393	−1.183 (0.210)**	0.306
Fairness	−0.150 (0.234)	0.861	0.227 (0.283)	1.255	0.045 (0.250)	1.046	0.725 (0.254)**	2.065	−0.875 (0.217)**	0.417
	**Profile 2 vs. Profile 4**		**Profile 3 vs. Profile 4**		**Profile 1 vs. Profile 3**		**Profile 2 vs. Profile 3**		**Profile 1 vs. Profile 2**	
	**Coef. (SE)**	**OR**	**Coef. (SE)**	**OR**	**Coef. (SE)**	**OR**	**Coef. (SE)**	**OR**	**Coef. (SE)**	**OR**
Sex (women)	−0.654 (0.355)	0.520	0.102 (0.375)	1.107	−1.297 (0.314)**	0.273	−0.756 (0.298)*	0.470	−0.541 (0.307)	0.582
Age	0.038 (0.192)	1.039	−0.155 (0.199)	0.857	0.468 (0.155)**	1.596	0.193 (0.158)	1.213	0.275 (0.143)	1.317
Org. tenure	−0.306 (0.178)	0.736	−0.121 (0.186)	0.886	−0.268 (0.150)	0.765	−0.185 (0.163)	0.831	−0.082 (0.146)	0.921
Education	−0.374 (0.177)*	0.688	−0.140 (0.166)	0.869	−0.402 (0.133)**	0.669	−0.234 (0.148)	0.792	−0.168 (0.134)	0.845
Rel. Status	0.134 (0.443)	1.143	0.548 (0.552)	1.731	−0.904 (0.402)*	0.405	−0.415 (0.410)	0.661	−0.489 (0.323)	0.613
#Children	−0.033 (0.180)	0.967	0.113 (0.179)	1.120	0.073 (0.134)	1.076	−0.147 (0.142)	0.864	0.220 (0.126)	1.246
Workload	−0.373 (0.203)	0.689	0.225 (0.214)	1.252	−0.151 (0.172)	0.860	−0.598 (0.183)**	0.550	0.447 (0.174)**	1.564
Control	−0.417 (0.204)*	0.659	−0.593 (0.246)*	0.553	0.383 (0.199)	1.467	0.176 (0.218)	1.192	0.207 (0.200)	1.230
Community	−0.784 (0.213)**	0.457	−0.503 (0.236)*	0.605	−0.681 (0.191)**	0.506	−0.281 (0.197)	0.755	−0.399 (0.173)*	0.671
Fairness	−0.498 (0.242)*	0.608	−0.680 (0.246)**	0.507	−0.195 (0.212)	0.823	0.182 (0.237)	1.199	−0.377 (0.213)	0.686

*p < 0.05; **p < 0.01; SE, Standard error of the coefficient; OR, Odds ratio; Sex (0 male, 1 female); Relationship status (0 single, 1 couple); #children: Number of dependent children living at home; workload, control, community, and equity are estimated from factor scores with a mean of 0 and a standard deviation of 1; age, tenure, and educational level were standardized prior to analysis; coefficients and OR reflect the effects of predictors on the likelihood of membership in the first listed profile relative to the second listed profile; Profile 1: Globally Unsatisfied; Profile 2: Globally Satisfied in Personal Life with High Autonomy; Profile 3: Globally Satisfied in Personal Life with Low Autonomy; Profile 4: Globally Satisfied at Work with High Relatedness; and Profile 5: Globally Satisfied.

#### Job characteristics

Individuals with higher workload perceptions presented a greater likelihood of membership into the *Globally Unsatisfied* (1) and *Globally Satisfied in Personal Life with Low Autonomy* (3) profiles over the *Globally Satisfied* (5) and *Globally Satisfied in Personal Life with High Autonomy* (2) profiles. These results support Hypothesis 2a, which posits that perceptions of high workload should increase the likelihood of membership in profiles mainly corresponding to the *Impoverished* and *Work*- and *Personal Life-Fulfilled* scenarios. When favorable job characteristics (job control, fairness, and sense of community) are considered, all three job resources were related to a greater likelihood of membership into the *Globally Satisfied at Work with High Relatedness* (4) profile over the *Globally Satisfied in Personal Life with Low Autonomy* (3) and *Globally Satisfied* (5) profiles. Job control and sense of community were also related to a greater likelihood of membership into the *Globally Satisfied at Work with High Relatedness* (4) profile compared to the *Globally Satisfied in Personal Life with High Autonomy* (2) profile, whereas sense of community and fairness were related to a greater likelihood of membership into the *Globally Satisfied at Work with High Relatedness* (4) profile over the *Globally Unsatisfied* (1) profile. Sense of community was also associated with a greater likelihood of membership into the *Globally Satisfied in Personal Life with High Autonomy* (2)*, Globally Satisfied in Personal Life with Low Autonomy* (3), and *Globally Satisfied* (5) profiles over the *Globally Unsatisfied* profile (1). Although these results suggest a beneficial work specific effect that is shared across job resources, and more generalized perhaps for the sense of community, they are fairly consistent with Hypothesis 2b which posits that perceptions of job resources increase the likelihood of membership in profiles mainly corresponding to the *Enriched* and *Work*- and *Personal Life-Fulfilled* scenarios. More specifically, the benefits of job resources are more pronounced in the profile characterized by higher global levels of need satisfaction at work with high specific levels of domain-general relatedness need satisfaction.

#### Individual characteristics

Addressing our research question, the results did reveal some noteworthy associations between individual characteristics and the likelihood of profile membership.^3^ First, being a woman increased the likelihood of membership into the *Globally Satisfied* (5), *Globally Satisfied at Work with High Relatedness* (4), and *Globally Satisfied in Personal Life with Low Autonomy* (3) profiles over the *Globally Unsatisfied* (1) profile. It also increased the likelihood of membership into the *Globally Satisfied* (5) over the *Globally Satisfied in Personal Life with High Autonomy* (2) and *Globally Satisfied in Personal Life with Low Autonomy* (3) profiles. This suggests that women are more likely to have their basic needs globally fulfilled at work and in personal life.

Older participants were more likely to belong to the *Globally Satisfied* (5) or *Globally Unsatisfied* (1) profiles than the *Globally Satisfied in Personal Life with Low Autonomy* (3) profile. In contrast, longer organizational tenure increased the likelihood of membership into the *Globally Satisfied at Work with High Relatedness* (4) profile over the *Globally Unsatisfied* (1) profile. This suggests a higher quality of relationships with members of the organization. The effects of education level were more widespread. Higher education was related to a greater likelihood of membership into the *Globally Satisfied in Personal Life with Low Autonomy* (3) and *Globally Satisfied at Work with High Relatedness* (4) profiles than into the *Globally Unsatisfied* (1) and *Globally Satisfied* (5) profiles, and of membership into the *Globally Satisfied at Work with High Relatedness* (4) profile than into *Globally Satisfied in Personal Life with High Autonomy* (2) profile.

Being in a couple (vs. single) increased the likelihood of membership into the *Globally Satisfied* (5) and *Globally Satisfied in Personal Life with Low Autonomy* (3) profiles compared to the *Globally Unsatisfied* (1) profile. In contrast, the number of dependent children living at home was associated with greater likelihood of membership into the *Globally Unsatisfied* (1) and *Globally Satisfied in Personal Life with Low Autonomy* (3) profiles, compared to the *Globally Satisfied* (1) profile.

### Need satisfaction profiles: organizational and individual outcomes

The results from the comparisons of outcome levels across profiles are reported in Table 4.

**Table 4:** Associations between profile membership and outcomes.

	Profile 1 M [CI]	Profile 2 M [CI]	Profile 3 M [CI]	Profile 4 M [CI]	Profile 5 M [CI]	Summary of statistically significant differences
Job satisfaction	−0.475 [−0.585; −0.365]	0.063 [−0.047; 0.173]	−0.322 [−0.455; −0.189]	0.689 [0.583; 0.795]	0.136 [0.005; 0.267]	1 = 3 < 2 = 5 < 4
Turnover intentions	0.076 [−0.040; 0.192]	−0.037 [−0.149; 0.075]	0.325 [0.176; 0.474]	−0.409 [−0.507; −0.311]	0.023 [−0.118; 0.164]	4 < 1 = 2 = 5 < 3
Psychological distress	0.450 [0.328; 0.572]	−0.351 [−0.443; −0.259]	0.344 [0.203; 0.485]	−0.258 [−0.380; −0.136]	−0.313 [−0.431; −0.195]	2 = 4 = 5 < 1 = 3
Vitality	−0.507 [−0.636; −0.378]	0.109 [0.009; 0.209]	−0.102 [−0.235; 0.031]	0.183 [0.061; 0.305]	0.572 [0.468; 0.676]	1 < 3 < 2 = 4 < 5
Absences (occurrence)	2.503 [2.215; 2.791]	2.011 [1.762; 2.260]	2.937 [2.565; 3.309]	1.542 [1.281; 1.803]	2.580 [2.215; 2.945]	4 < 2 < 1 = 3 = 5
Absences (days missed)	10.695 [8.318; 13.072]	9.651 [7.566; 11.736]	9.804 [7.295; 12.313]	3.250 [2.566; 3.934]	9.367 [6.870; 11.864]	4 < 1 = 2 = 3 = 5
Work injury (incidents)	0.105 [0.056; 0.154]	0.047 [0.018; 0.076]	0.072 [0.027; 0.117]	0.004 [−0.006; 0.014]	0.014 [−0.006; 0.034]	4 < 2 < 1; 4 = 5 < 1 = 3; 2 = 3; 2 = 5
Work injury (days missed)	1.018 [0.259; 1.777]	0.929 [0.218; 1.640]	0.424 [−0.054; 0.902]	0.018 [−0.017; 0.053]	0.084 [−0.036; 0.204]	4 = 5 < 1 = 2; 1 = 2 = 3; 3 = 4 = 5
Absenteeism (days)	10.273 [7.255; 13.291]	7.876 [5.824; 9.928]	10.491 [6.812; 14.170]	3.106 [2.447; 3.765]	11.307 [7.069; 15.545]	4 < 1 = 2 = 3 = 5
Presenteeism	21.405 [14.357; 28.453]	13.330 [9.032; 17.628]	17.993 [1.915; 25.071]	4.194 [3.441; 4.947]	16.330 [9.537; 23.123]	4 < 1 = 2 = 3 = 5
Self-rated job performance	2.567 [2.455; 2.679]	2.163 [2.065; 2.261]	2.495 [2.366; 2.624]	2.013 [1.901; 2.125]	2.123 [2.003; 2.243]	2 = 5 < 1 = 3; 4 < 2 < 1 = 3; 4 = 5

M, Mean; CI: 95% Confidence interval; indicators of job satisfaction, turnover intentions, psychological distress, and vitality are estimated from factor scores with a mean of 0 and a standard deviation of 1; OR: Indicators obtained from official organizational records; SR: Self-reported single items; Profile 1: Globally Unsatisfied; Profile 2: Globally Satisfied in Personal Life with High Autonomy; Profile 3: Globally Satisfied in Personal Life with Low Autonomy; Profile 4: Globally Satisfied at Work with High Relatedness; and Profile 5: Globally Satisfied.

#### Organizational outcomes

With respect to job attitudes, the results showed that job satisfaction was the highest for the *Globally Satisfied at Work with High Relatedness* (4) profile, followed equally by the *Globally Satisfied* (5) and *Globally Satisfied in Personal Life with High Autonomy* (2) profiles, and then equally by the *Globally Unsatisfied* (1) and *Globally Satisfied in Personal Life with Low Autonomy* (3) profiles. Turnover intentions were the highest for the *Globally Satisfied in Personal Life with Low Autonomy* (3) profile and the lowest for the *Globally Satisfied at Work with High Relatedness* (4) profile, with the remaining profiles falling in between (*Globally Unsatisfied*, *Globally Satisfied in Personal Life with High Autonomy,* and *Globally Satisfied*).

As for job behaviors, self-reported levels of job performance were higher for the *Globally Satisfied* (5) and *Globally Satisfied in Personal Life with High Autonomy* (2) profiles than the *Globally Unsatisfied* (1) and *Globally Satisfied in Personal Life with Low Autonomy* (3) profiles, and for the *Globally Satisfied at Work with High Relatedness* (4) profile than the *Globally Satisfied in Personal Life with High Autonomy* (2) and *Globally Satisfied* (5) profiles. Results from official reports of the number of days missed due to absenteeism matched the results on self-reported days missed due to absenteeism and days of presenteeism, being lower in the *Globally Satisfied at Work with High Relatedness* (4) profile than in all other profiles. Interestingly, official reports of absence occurrence (vs. days missed) lead to more nuanced results, being lower in the *Globally Satisfied at Work with High Relatedness* (4) profile than in all other profiles, but also lower in the *Globally Satisfied in Personal Life with High Autonomy* (2) profile than in the remaining profiles (*Globally Unsatisfied*, *Globally Satisfied in Personal Life with Low Autonomy*, and *Globally Satisfied*).

The results from official reports of work injuries showed higher accident incidents for the *Globally Unsatisfied* (1) profile than the *Globally Satisfied in Personal Life with High Autonomy* (2) profile, which showed higher occurrence than the *Globally Satisfied at Work with High Relatedness* (4) profile. Accident incidents were also higher in the *Globally Unsatisfied* (1) and *Globally Satisfied in Personal Life with Low Autonomy* (3) profiles than in the *Globally Satisfied at Work with High Relatedness* (4) and *Globally Satisfied* (5) profiles. As for absenteeism, official reports of the days missed due to work injuries afforded less precision, simply higher for the *Globally Unsatisfied* (1) and *Globally Satisfied in Personal Life with High Autonomy* (2) profiles than the *Globally Satisfied at Work with High Relatedness* (4) and *Globally Satisfied* (5) profiles.

These results are consistent with Hypothesis 3a, which posits that profiles mainly corresponding to the *Enriched* and *Work*- and *Personal Life-Fulfilled* scenarios will be associated with favorable job attitudes and behaviors. They further suggest that misaligned global profiles (globally satisfied predominantly at work or in personal life) with high specific need satisfaction in terms of autonomy or relatedness (reflecting a positive imbalance of specific needs) are even more strongly associated with favorable organizational outcomes.

#### Individual outcomes

In support of Hypothesis 3b, the results showed higher levels of vitality in the *Globally Satisfied* (5) profile than in all other profiles, followed equally by the *Globally Satisfied at Work with High Relatedness* (4) and *Globally Satisfied in Personal Life with High Autonomy* (2) profiles, which were followed in turn by the *Globally Satisfied in Personal Life with Low Autonomy* (3) profile and the *Globally Unsatisfied* (1) profile. Similarly, levels of psychological distress were lower in the *Globally Satisfied* (5), *Globally Satisfied at Work with High Relatedness* (4), and *Globally Satisfied in Personal Life with High Autonomy* (2) profiles than in the *Globally Unsatisfied* (1) and *Globally Satisfied in Personal Life with Low Autonomy* (3) profiles.

## Discussion

This study enriches our understanding of psychological need satisfaction at the interface of work and personal life via the conceptual elaboration and empirical examination of a comprehensive typology. Our results revealed five profiles, consistent with four of our five scenarios: *Enriched, Impoverished, Work-Fulfilled, and Personal Life-Fulfilled*. Unfortunately, no profile corresponding to the *Middling* scenario was observed. In addition to perceptions of job characteristics, individual characteristics were also found to predict membership into these distinct need satisfaction profiles.

### Theoretical contributions

#### A typology of need satisfaction at work and in personal life

The proposed typology is based on the assumption, inspired by research on work–personal life interface (Frone et al., 1992; Greenhaus and Powell, 2006), that the extent to which individual basic psychological needs will be satisfied can differ across domains. This assumption is supported by our results, which indicate that global need satisfaction remains somewhat independent across these two domains. Thus, individuals might manage to globally fulfill their psychological needs at work but not necessarily in their personal life, and *vice-versa*. Highlighting the need to explore in greater depth the domain-specific nature of need satisfaction across important life contexts, this tends to refute the one-size-fits-all approach that some SDT-based studies have advocated using global (context-free) measures to assess overall need satisfaction or in life as a whole (see for a review, Tóth-Király et al., 2020).

The identification of distinct configurations of need satisfaction at work and in personal life offers significant empirical support, albeit preliminary, to the proposed typology. Importantly, the five identified profiles correspond to four of the five proposed scenarios. Two profiles (*Globally Satisfied* and *Globally Unsatisfied*) correspond to two of the three proposed aligned scenarios (*Enriched* and *Impoverished*), whereas the remaining profiles (*Globally Satisfied at Work with High Relatedness*, *Globally Satisfied in Personal Life with High Autonomy*, and *Globally Satisfied in Personal Life with Low Autonomy*) correspond to the two misaligned scenarios (*Work-Fulfilled* and *Personal Life-Fulfilled*). Interestingly, the two profiles corresponding to the *Personal Life-Fulfilled* scenario appear to capture a marked imbalance in the satisfaction of the specific need for autonomy (high vs. low specific levels) relative to the other specific needs, and the nature of this imbalance (i.e., positive vs. negative) appears to play a clear role in driving outcome associations. In contrast, in the profile corresponding to the *Work-Fulfilled* scenario, imbalance seemed to be limited to the need for relatedness, and strictly positive in the present study.

These observations underscore the importance to consider the alignment of global need satisfaction across the work and personal life domain, as well as the degree, and nature, of imbalance in the satisfaction of each specific need in relation to the global level among profiles exhibiting misaligned global levels of need satisfaction across domains. Indeed, and contrary to our expectations, none of the aligned profiles (*Globally Satisfied* and *Globally Unsatisfied*) displayed imbalanced levels of specific need satisfaction. Although these results are consistent with the notion of resources caravans (Hobfoll, 2002), positive and negative forms of imbalance in the satisfaction of specific needs remain possible. This would be the case of individuals for whom specific needs are fulfilled to a slightly higher (or lower) extent than their global levels of need satisfaction. For example, a business owner-manager might be able to benefit from a particularly fulfilling work and personal life, but also experience particularly low specific levels of autonomy need satisfaction due to lack of time or resources (e.g., staff) to invest in other meaningful activities (e.g., coaching his/her kids in sports or taking other active role in the community). However, because this study is the first empirical examination of the typology, future studies could potentially identify other profiles depending on the alignment of the levels of global need satisfaction at work and in personal life and the configuration of the specific satisfied needs. In particular, it would be interesting to better document whether other types of imbalance would be identified (i.e., whether aligned profiles can display imbalance, whether autonomy imbalance is limited to the *Personal Life-Fulfilled* scenario, and whether relatedness imbalance is limited to positive manifestations associated with the *Work-Fulfilled* scenario). Likewise, it would be important for future studies to expand upon our results by simultaneously considering other need states (frustration and unfulfillment) across the work and personal life domain. This could help to delve further into each scenario, including the Middling, for which no profile was identified in our study sample, and which contrasts with prior-centered research on need satisfaction (Gillet et al., 2019; Huyghebaert-Zouaghi et al., 2020; Tóth-Király et al., 2020). In these latter studies, Normative profiles characterized by close to average level of need states were observed. In relation to the proposed typology, it could be except that globally satisfied at work and in personal life with mixed specific need states (i.e., different levels of specific need fulfillment, satisfaction and/or frustration) profiles reflect the make-up of employees belonging to the Middling scenario.

#### The role of job and individual characteristics

This study sheds new light on the predictors of need satisfaction profiles at the interface of work and personal life. Although based on a limited number of predictors, the results tend to delimit the contextualized (vs. overall) contribution of certain workplace and individual characteristics to membership of distinct need satisfaction profiles. Although some job resources (job control, fairness, and sense of community) appeared to facilitate the likelihood of membership in profiles in which the work domain predominates (*Globally Satisfied at Work with High Relatedness*), a sense of community at work tended to overlap with personal life, and was found to be associated with membership into profiles in which personal life predominates (*Globally Satisfied in Personal Life with High* or *Low Autonomy*) or in which the two domains are aligned (e.g., *Globally Satisfied* or *Globally Unsatisfied*). These results strengthen the premise that some resources are instrumental for achieving job objectives (Schaufeli and Bakker, 2004) while also contributing to enriching individual lives more generally. However, similar to a low sense of community at work, a heavy workload hinders global levels of need satisfaction both at work and in personal life. By limiting the psychological nutrients that are essential to both domains, a heavy workload would strengthen the health impairment effect proposed by the JD-R model (Bakker and Demerouti, 2017). These findings urge us to reconsider the specificity of other demands (e.g., challenge or hindrance stressors; Podsakoff et al., 2007) that might also act on need satisfaction simultaneously at the interface of work and personal life.

A compelling feature of our study is that certain individual characteristics appeared to be naturally associated with profile membership in terms of need satisfaction at work and in personal life. These individual differences have been generally neglected in the SDT literature, possibly because SDT assumes that the needs are universal (Ryan and Deci, 2017). However, this does not prevent need satisfaction levels from varying across individuals, as evidenced by our results. In our sample, women reported higher global levels of need satisfaction, at work and in personal life. This is surprising, as some research suggests that women might find it more difficult to balance the demands of professional and family life, and might be more subject to job-related strain reactions (e.g., Shirom et al., 1999). These findings appear more in line with the enhancement, rather than scarcity, hypothesis of role theory (Marks, 1977; Chapman et al., 1994), according to which multiple roles enrich one’s resources.

Furthermore, older employees tended to belong more frequently to profiles displaying an aligned configuration across domains. Although this effect appeared to occur at both extremes (i.e., in relation to the *Globally Satisfied* and *Globally Unsatisfied* profiles), this result does suggest that aging might favor the emergence of a greater level of alignment in the extent to which one’s basic needs are fulfilled across domains. In contrast, organizational tenure and education levels appeared to favor a predominantly *Work-Fulfilled* profile, in which the specific level of relatedness need satisfaction is particularly high (*Globally Satisfied at Work with High Relatedness* profile). This result is consistent with the idea that more tenured and educated employees might have developed stronger relational ties in their workplaces. Moreover, although living as a couple appeared to foster a *Globally Satisfied* profile, having dependent children living at home rather seemed to favor a *Globally Unsatisfied* profile. Interestingly, both characteristics (couple and children) also contributed to membership into the *Globally Satisfied in Personal Life with Low Autonomy* profile, consistent with the idea that family life can be fulfilling, but also impede one’s autonomy. Although it would be premature to draw definitive conclusions about the contribution of individual characteristics to the profiles, these results suggest that future investigations should be more systematic on this issue. Such an approach could inform the development and implementation of human resource (HR) strategies that are more consistent with the realities of diverse employees.

#### The importance of need satisfaction profiles from an outcomes perspective

This study extends the knowledge of the potential effects of profiles of psychological need satisfaction at work and in personal life on individual and organizational outcomes. According to the proposed typology, the results considerably nuance the notion of a balanced satisfaction of psychological needs, as proposed by Sheldon and Niemiec (2006). Indeed, our results show that, what appears to be particularly important for individual well-being is the amount of global need satisfaction, *when work and personal life are aligned*. In contrast, when both domains appear to be misaligned, it is the positive or negative nature of the imbalance among the specific needs that becomes critical. In particular, and contrary to Sheldon and Niemiec (2006) original proposal, a positive imbalance seemed to be far more desirable than a negative imbalance.

More specifically, among the aligned profiles, the *Globally Unsatisfied* profile (*Impoverished*) was found to be associated with some of the least desirable outcome levels observed in the present study, whereas the *Globally Satisfied* profile (*Enriched*) was found to be associated with some of the most desirable outcome levels. Although these results concur with SDT predictions (Deci et al., 2017) and with prior variable-centered (Van den Broeck et al., 2016; Gillet et al., 2020a) and person-centered (Gillet et al., 2019, 2020b) research results, they still add to the knowledge by demonstrating that these predictions generalize to a range of self-reported and objectively-measure outcomes, as well as to the joint consideration of the work and personal life domains. It is noteworthy that the *Globally Unsatisfied* profile stands apart from the other profiles by its consistent associations with work behaviors that are tied to individual health (presenteeism, absenteeism, and injuries). Low global levels of need satisfaction at work and in personal life therefore appear to contribute to withdrawal behaviors and to increase injury proneness at work.

In addition, our results also reveal a rich pattern of domain-specific outcomes associated with the misaligned profiles, which seems to favor the work domain as well as positive forms of imbalance. When one’s psychological needs are globally satisfied at work (*Globally Satisfied at Work with High Relatedness* profile), the predominant specific satisfaction of one need over the others (relatedness in this case) was found to contribute to a range of favorable outcomes, sometimes even more favorable than those observed in the *Globally Satisfied* profile (i.e., higher job satisfaction, lower turnover intentions, higher self-rated job performance, lower absenteeism, and lower presenteeism). Likewise, when one’s psychological needs are globally met in the personal life domain, and accompanied by a positive imbalance in the satisfaction of one need over the other (autonomy in this context, as captured in the *Globally Satisfied in Personal Life with High Autonomy* profile), outcome levels are generally on par with those observed in the *Globally Satisfied* profile. In contrast, when similar global levels of need satisfaction are accompanied by a negative imbalance in the satisfaction of a specific need (autonomy in this context, as captured in the *Globally Satisfied in Personal Life with Low Autonomy* profile), the desirability of outcome levels decreases substantially, to reach a level comparable to that observed in the *Globally Dissatisfied* profile. This pattern of results involving the specific need for autonomy thereby reinforces the idea that one should not only consider the satisfaction of each need taken in isolation (such as the salient role of the need for autonomy; Trépanier et al., 2015b), but that it is even more important to consider each need within the overarching configuration of needs occurring within a profile. Indeed, this last approach makes it possible to consider how the nature (positive vs. negative) of imbalance in the satisfaction of each need is likely to influence key individual and organizational outcomes beyond individuals’ global levels of need satisfaction.

These results further suggest that when there is a predominance of global levels of satisfaction at work or in personal life, any marked elevation (i.e., positive imbalance) in the satisfaction of specific need (*relatedness* for the *Work-Fulfilled* profile and *autonomy* for the *Personal Life-Fulfilled* profile), could compensate for the lower level of satisfaction across all three needs in one specific life domain. In contrast, any markedly reduction (i.e., negative imbalance) in the satisfaction of a specific need (*autonomy* for the *Personal Life-Fulfilled* profile) is likely to offset any gains afforded by a higher global level of need satisfaction in a specific life domain. Although we can only speculate on the relative contribution of each specific factor, it would be informative to delve deeper into the phenomenological nature of the specific needs.

In a broader sense, and although the present study mainly relied on job characteristics, the findings offer some insight into the work–personal life interface research. They point to an additional psychological path whereby the work context can simultaneously enrich and impoverish personal and professional life as well as overall well-being. Accordingly, employees in the *Globally Satisfied in Personal Life with Low Autonomy* profile could be especially vulnerable to a substantial work-life interference (Rothbard, 2001), which might be anchored in a lack of autonomy to make decisions regarding conflictual demands of both life domains, and potentially triggered by increases in workload or in personal life demands. Future studies could seek to improve our understanding of the domain-specific outcomes and predictors of these profiles, especially in personal life (e.g., life satisfaction, couple satisfaction, community involvement). It would also be useful to examine the contribution of the regulation processes and psychological mechanisms that underlie the proposed typology. For example, would low global need satisfaction levels at work or in personal life inevitably lead to mixed behavioral regulations (autonomous and controlled) and accommodation or compensatory strategies? Depending on the case, are they domain-specific, or could they be transposed to another domain?

### Limitations and future directions

This study includes limitations. First, despite the objective data (absence and injuries), which led to results similar to those obtained via self-reports, this study remains mainly based on self-report measures. This raises the possibility of social desirability and self-assessment bias. Future studies should include multiple sources of data (e.g., partner, friends) and a broader range of objectives (e.g., physiological indicators of stress) to increase the scope of the results. In addition, as the reliability of the need satisfaction S-factors used in our analyses were either at the lower limit of acceptability, or well below that limit. Although this low reliability is unlikely to have interfered with the profile estimation process, it does limit our ability to identify profiles characterized by an imbalanced configuration across all three needs. It would thus seem critical for future research to rely on the full version of the Basic Need Satisfaction Scale or other instruments with established evidence of psychometric properties. Second, we used a cross-sectional design, which does not allow establishing the temporal stability of the profiles or definitively determining causal relations between the variables. Although studies provide support for some of the proposed associations (e.g., Trépanier et al., 2015b; Huyghebaert et al., 2018), we cannot exclude the possibility of reciprocal or inverse relations between certain variables (Cole and Maxwell, 2003). It is plausible that individual attitudes and behaviors could have acted on the need satisfaction profiles. Future studies should examine the nature of the relations using instruments designed to substantiate the temporal ordering of the observed associations. Third, although we relied on a proven theoretical perspective to determine the choice of predictors likely to act on profile membership, our analysis remains based on a limited number of theoretical antecedents, particularly with respect to personal life. Whereas the independence of global levels of need satisfaction between work and personal life has been established, further studies are needed to extend the understanding of the predictors, particularly in the personal life domain (e.g., personal life and family stressors, role conflict, enrichment; Frone et al., 1992; Casper et al., 2018). Likewise, it would be important to expand upon our results by considering additional indicators of functioning specific to personal life (e.g., personal life satisfaction, family involvement, proactive investment in leisure activities). Finally, with respect to the results generalization, our study addresses a sample of employees working for a single organization in one European country. Our results should be replicated in samples of employees from various occupations, industries, and cultures.

### Managerial implications

Despite these limitations, our results have new managerial implications for the promotion of favorable job attitudes and behaviors and overall employee well-being. From an organizational perspective, the identification of psychological need satisfaction profiles encompassing employees’ work and personal life offers promising tools for personnel managers who seek to improve prescribed HR practices. Consistent with the fashion of adopting innovative individual-centered HR practices that are said to be more humane (Jiang et al., 2012), our results suggest that employees more liable to present adaptive profiles of psychological need satisfaction and those who are at risk for certain professional and/or personal problems could both be identified for purposes of intervention seeking to help the former maintain this desirable scenario and the latter move away from their undesirable scenario. Armed with this understanding, managers would be equipped to implement policies, measures, and actions better connected to the psychological realities of their employees, for example, by offering enabling vs. enclosing work-life policies (Bourdeau et al., 2019).

The present typological approach promises substantial benefits by promoting both individual (higher overall well-being) and organizational (higher job satisfaction and performance, and lower turnover intentions) gains, while cutting organizational costs (absenteeism, presenteeism, and injuries). Given that the basic psychological needs are largely invariant across individuals and cultures (Deci et al., 2001; Chen et al., 2015), it is up to organizations to instill practices that enable a more harmonized satisfaction of these needs across employees with diverse profiles. Thus, strategies that simultaneously account for need satisfaction at work and in personal life could be designed and deployed to foster both work–life balance, but also improved organizational functioning. To this end, our results suggest that changes designed to reduce employees’ workload and to increase their sense of community could be particularly useful. For instance, workload could be limited at the organizational level by stating clear segmentation norms and encouraging balanced and healthier lifestyles (Kreiner, 2006). Workload could also be reduced at the individual level through coaching or counseling (e.g., developing new habits and replacing one’s old malfunctioning behaviors; Van Gordon et al., 2017). In addition, enriching and strengthening social bonds in and outside the work environment should constitute effective levers of intervention. At the organizational level, managers might promote greater fairness and autonomy in the applications of policies to reinforce the supportive culture. Social events might also include family members in efforts to nurture personal and professional relationships and communities.

Moreover, organizations would benefit by considering individual characteristics (age, sex, organizational tenure, and family situation) and by inquiring into environmental aspects of the workplace such as workload and resource availability. This would be especially relevant for managers, who play a leadership role. Because to their inner qualities and outward behaviors, authentic and transformational managers can shape employees’ attitudes and behaviors by influencing their perceptions of the workplace (Piccolo and Colquitt, 2006; Fernet et al., 2015) and by ensuring that their psychological needs are met (Kovjanic et al., 2012). In parallel, technology-based interventions could be developed to raise awareness of employees’ need satisfaction. Gradito Dubord et al. (2022) recently found that the use of an online platform on signature strengths (i.e., capacity for an authentic and energizing way of behaving, thinking or feeling) facilitates need satisfaction and fosters well-being.

## Conclusion

This study adds to the understanding of psychological need satisfaction at the interface of work and personal life. It lays the foundation for a typology that may become the starting point for future theory development and research. Our results identify five profiles of need satisfaction that uphold the conceptual and empirical distinctness of four out of five of our typology-based scenarios. Our results also showed that the amount of global need satisfaction is primordial to consider when work and personal life are aligned, whereas the amount and nature of the imbalance among specific need satisfaction levels are equally important to consider when there is misalignment across domains. In addition, our results highlight certain organizational and individual predictors that could serve as intervention levers to promote more adaptable profiles. The outcomes would include more positive attitudes and behaviors at work and improved overall well-being for individuals.

## Data availability statement

The raw data supporting the conclusions of this article will be made available by the authors, without undue reservation.

## Ethics statement

The requirement of ethical approval was waived by Jack Welch College of Business and Technology, Sacred Hearth University, Luxembourg for the studies involving humans. The study was conducted in accordance with the local legislation and institutional requirements, which do not require assessment for minimal risk studies. The participants provided their written informed consent to participate in this study.

## Author contributions

CF: conceptualization, methodology, writing—original draft, writing—review and editing. AM and NG: conceptualization, methodology, formal analysis, writing—original draft, writing—review and editing. MM: conceptualization, methodology, data collection, writing—original draft, writing—review and editing. SA: conceptualization, writing—review and editing. All authors contributed to the article and approved the submitted version.

## Funding

Preparation of this paper was supported by grants from the Fonds de Recherche du Québec – Société et Culture (2019-SE1-252542) and the Social Science and Humanity Research Council of Canada (435-2018-0368).

## Conflict of interest

The authors declare that the research was conducted in the absence of any commercial or financial relationships that could be construed as a potential conflict of interest.

## Publisher’s note

All claims expressed in this article are solely those of the authors and do not necessarily represent those of their affiliated organizations, or those of the publisher, the editors and the reviewers. Any product that may be evaluated in this article, or claim that may be made by its manufacturer, is not guaranteed or endorsed by the publisher.

## Footnotes

^1^Although these values are within the lowest range of acceptability, they are each based on only three items. Knowing that reliability is negatively impacted by the number of items (e.g., Streiner, 2003), these coefficients would be larger if they were based on eight equivalent items (ω adjusted using Spearman-Brown prophecy formula to eight equivalent items = 0.69–0.86). Yet, this observation reinforces the importance of relying on analyses providing some correction for unreliability (factor scores in the present study). More worrisome, however, are the very high factor correlations observed between the need satisfaction subscales in each domain (work: *r* = 0.82–0.96; personal life: *r* = 0.88–0.89), but not across domains (*r* = 0.10–0.34). This observation reinforces the value of a bifactor representation.

^2^It is important to note that, although the LPA conducted in the present study relied on factor scores saved from this final solution, including the competence S-factor, these analyses were also replicated without this S-factor and converged on an identical solution (results available upon request). This observation is in accordance with the observation that the competence S-factor did not differ meaningfully across profiles.

^3^Conducting the analyses with, or without, the demographic predictors resulted in the same conclusions regarding the role of the job characteristics.
