# Supplementary material for: Psychological need satisfaction across work and personal life: an empirical test of a comprehensive typology
Source: Front Psychol. 2023 Sep 6;14:1216450. doi: 10.3389/fpsyg.2023.1216450 (PMC10512304; doi:10.3389/fpsyg.2023.1216450)
Supplement: Supplementary file 1 [file Data_Sheet_1.docx]

**Online Supplemental Materials for:**

Psychological Need Satisfaction across Work and Personal Life:

An Empirical Test of a Comprehensive Typology

Authors’ note:

These online technical appendices are to be posted on the journal website and hot-linked to the manuscript. If the journal does not offer this possibility, these materials can alternatively be posted on one of our personal websites (we will adjust the in-text reference upon acceptance).

We would also be happy to have some of these materials brought back into the main manuscript, or included as published appendices if you deem it useful. We developed these materials to provide additional technical information and to keep the main manuscript from becoming needlessly long.

**Preliminary Measurement Models: Need Satisfaction across Work and Personal Life**

**Analyses**

All analyses were conducted using Mplus 8.3 (Muthén & Muthén, 2019) maximum likelihood estimator robust to non-normality (MLR). Due to the online data collection mode, there was no missing data. In a first stage of analyses, we contrasted a series of alternative measurement models to identify the most optimal representation of participants’ need satisfaction ratings across work and personal life. As a baseline comparison model, we first considered an a priori representation of participants’ responses encompassing six correlated confirmatory factor analytic (CFA) factors representing autonomy, competence and relatedness need satisfaction across work and personal life. This solution thus included a total of six correlated domain-specific need satisfaction factors (work-autonomy, work-competence, work-relatedness, personal life-autonomy, personal life-competence, and personal life-relatedness).

Then, based on mounting evidence suggesting that a bifactor representation is most suitable for need satisfaction ratings across life domains (e.g., Garn et al., 2018; Gillet et al., 2019, 2020), we contrasted an a priori six factor CFA solution to four alternative bifactor solutions. First, we incorporated two distinct bifactor models (one per domain) into a single solution, where one G-factor and three orthogonal S-factors were specified for each domain, and allowed to correlate across domains. This first solution thus includes two correlated domain-specific G-factors (work satisfaction and personal life satisfaction) and six domain-specific S-factors (work-autonomy, work-competence, work-relatedness, personal life-autonomy, personal life-competence, and personal life-relatedness) correlated across domains but not within domain^[[1]](#footnote-1)^. In a second solution (e.g., Tóth-Király et al., 2020), a single domain-general G-factor is specified to represent participants’ global levels of need satisfaction across domains, while the S-factors remain domain-specific (work-autonomy, work-competence, work-relatedness, personal life-autonomy, personal life-competence, and personal life-relatedness) and correlated across domains but not within domain. In a third solution, two correlated domain-specific G-factors (work satisfaction and personal life satisfaction) are estimated, but in this solution only three orthogonal domain-general S-factors are estimated (i.e., not differentiated across domains: general-autonomy, general-competence, and general-relatedness). Whereas the previous model assumed that global levels of satisfaction tend to be shared across domains, this third model rather assumes that global levels of need satisfaction can be differentiated across domains whereas their specific tendencies to deviate from this global level (autonomy, competence, and relatedness) tend to be shared across domains. For comparison purposes, we estimated a final model including one domain-general G-factor and three orthogonal domain-general S-factors. Across all models, a priori correlated uniquenesses between matching indicators of the factors utilized across domains were included to control for wording effects (e.g., Marsh, 2007; Marsh et al., 2013).

These alternative solutions were compared using the following sample-size-independent fit indices (Marsh et al., 2005): The Root Mean Square Error of Approximation (RMSEA), the Tucker-Lewis Index (TLI), and the Comparative Fit Index (CFI). Values smaller than .08 or .06 for the RMSEA, and values greater than .90 or .95 for the CFI and TLI respectively support acceptable and excellent model fit (Hu & Bentler, 1999; Marsh et al., 2005). Guidelines for nested model comparisons indicate that changes in CFI/TLI of at least .010, and changes in RMSEA of at least .015 can be taken to suggest a model fit differences across models (Chen, 2007; Cheung & Rensvold, 2002) which should be accompanied by an examination of parameter estimates. We also report omega (ω; McDonald, 1970) coefficients of composite reliability (Morin et al., 2020).

As a final verification, we relied on the final retained measurement models for tests of Differential Item Functioning (DIF) to verify that no measurement bias was introduce by the three linguistic versions of our questionnaire. To represent these three linguistic groups, two dummy variables were created, and used to predict item responses and scores on the factors through a Multiple Indicator Multiple Cause (MIMIC) approach (e.g., Morin et al., 2013). Three alternative models were contrasted. In the first, *null*, model, relations between the dummy variables and item responses, as well as between the dummy variables and the factors, were constrained to be zero. This model is consistent with a lack of differences across linguistic groups. In the second, *saturated* model, relations between the dummy variables and all item responses were freely estimated, while those between the dummy variables and the factors were still constrained to be zero. In the third, *invariant*, model, relations between the dummy variables and item responses were constrained by be zero, whereas those between the dummy variables and the factors were freely estimated. Comparison between the *null* and *saturated* models indicates whether the predictors have an impact on item responses. If this is the case, then the comparison between the saturated and invariant models indicates whether this influence can be entirely captured at the level of the factors (consistent with a lack of measurement biases) or whether additional effects need to be added to reflect the presence of DIF.

**Results**

The model fit statistics associated with the alternative measurement models estimated in the present study are reported in Table S1. These results first show that the a priori CFA model achieved an acceptable level of fit to the data. The parameter estimates from this model are reported in Table S2, and reveal autonomy and competence factors that are generally well-defined, with ω values of .696 for work autonomy, .636 for personal life autonomy, .612 for work relatedness, and .623 for personal life relatedness. However, the competence factors appeared to be weaker (ω = .450 at work and .518 in personal life), mainly due to the weak performance of the negatively-worded item from this scale (Item 1: “I do not feel very competent [at work]”). Negatively-worded items are known to create more ambiguity than positively-worded items, and thus often lead to lower reliability scores (Marsh, 1996). It was, however, not possible to simply remove that item from the study, as the presence of a locally non-identified specific factor (with 2 indicators) in the orthogonal bifactor models interfered with our ability to properly estimate these models. Although these values are within the lowest range of acceptability, it is important to reinforce the fact that they are based on only three items each. Knowing that reliability is negatively impacted by the number of items forming a scale (e.g., Streiner, 2003), it is noteworthy that these coefficients would be larger if they were based on eight equivalent items (ω adjusted using Spearman-Brown prophecy formula to eight equivalent items = .686 to .859). Yet, this observation reinforces the importance of relying on an approach providing a way to achieve some level of correction for unreliability in the estimation of the LPA models to be discussed later (i.e., factor scores in the present study). More worrisome, however, are the very high factor correlations observed between the need satisfaction subscales within each domain (work: *r* = .817 to .961; personal life: *r* = .884 to .889), but not across domains (*r* = .095 to .341). This observation reinforces the value of examining bifactor representations in order to obtain more differentiated estimates of global and specific need satisfaction components within each domain.

Although the next two models (including one or two global factors, and six domain-specific S-factors) resulted in an noteworthy increase in model fit (ΔCFI = +.016 to +.023; ΔTLI = +.013 to +.024; ΔRMSEA = -.004 to -.007), neither of those two solutions proved to be fully proper statistically due to the estimation of correlations between pairs of S-factors over 1.000 across domains, suggesting that the tendency to deviate from one’s global level of need satisfaction tends to be shared across domains (i.e., domain-general). This observation thus supported the relevance of pursuing alternative models including domain-general S-factors. In contrast, the correlation obtained between the two G-factors proved to be much smaller (*r* = .177), supporting the domain-specificity of global levels of need satisfaction. Hence, the Bifactor-CFA-3 solution, in which a single G-factor was used to represent levels of need satisfaction across domains, failed to reach an acceptable fit to the data.

In contrast, the last solution (Bifactor-CFA-4) was able to achieve a level of fit to the data comparable to that of the initial six-factor CFA model (ΔCFI = -.001; ΔTLI = -.006; ΔRMSEA = +.001) while converging on a fully proper solution not tainted by conceptual overlap between the factors. The results from this solution are reported in Table S3.

Despite the negatively worded item of the competence subscale, which still perform weakly in this solution and should be targeted for re-assessment in future studies, both G-factors appear well-defined (ω = .804 at work and .815 in personal life) and relatively independent (*r* = .159), supporting the domain-specificity of global need satisfaction ratings. Although the S-factors obtained as part of this solution appear to be weaker than their G-factors counterparts (ω = .486 for autonomy, .432 for relatedness, and .222 for competence), it is important to reinforce that both the autonomy and relatedness S-factors do retain some meaningful level of specificity anchored in a subset of items presenting construct-relevant variance left unexplained by the G-factors. Conversely, the competence S-factor appears to “vanish” once the variance explained by the G-factors is taken into account.

Yet, such observations are frequent with bifactor modeling, leading Morin et al. (2016) to reinforce that, whereas the observation of one or more well-defined G-factor(s) is critical to support the adequacy of a bifactor solution, it is not necessary for the S-factors to be equally well-defined. Morin et al. (2020) add that this observation simply suggests that the items associated with these specific factors only retain a limited amount of specificity once the variance explained by the global factor(s) is taken into account, which should not be taken to suggest that this minimal specificity is not relevant to consider. For this reason, they also reinforce the fact that typical interpretation guidelines for reliability cannot be directly applied to S-factors given that a bifactor model involves the division of the reliable variance present at the item level into two distinct factors. For this reason, many have argued that leniency (suggesting that values approaching .500 could be considered to be acceptable) was required for omega coefficients taken from bifactor models (Morin et al., 2020; Pereira et al., 2018). This suggests that the omega obtained for the autonomy (*ω* = .486) and relatedness (*ω* =.432) S-factors remain borderline acceptable, indicating that that some imbalance occurs for some participants on two of the S-factors (autonomy and relatedness) but far more rarely for the competence S-factor (*ω* =.222). Yet, such observations also reinforce the need to rely on analytical methods providing some degree of control for unreliability. It is finally important to reinforce that the observation of “vanishing” S-factors appears to be the norm in studies relying on a bifactor approach to need satisfaction measurement (Gillet et al., 2019, 2020a, 2020b). In the context of the present study, the results thus simply suggest that competence need satisfaction only retains negligible amounts of discrepancies or imbalance relative to global levels of need satisfaction.

Finally, the results from the analyses of DIF conducted as a function of the three linguistic are reported in Table S1. Although these results are consistent with the presence of group differences among all three linguistic groups (i.e., the fit of the saturated model is much higher than that of the null model), they also indicate that these differences are limited to scores on the factors (i.e., some groups score higher and lower on the factors) rather than at the level of item responses (i.e., the fit of the invariant model is does not differ from that of the saturated model, as shown by ΔCFI < .01, ΔTLI < .01, and ΔRMSEA < .015).

**Preliminary Measurement Models for the Predictors and Outcomes**

Preliminary analyses designed to verify the psychometric properties of the multi-item predictor and outcome measures were conducted using Mplus 8.3 (Muthén & Muthén, 2019) maximum likelihood estimator robust to non-normality (MLR). These analyses were conducted separately for the predictors and the outcomes, and model fit was assessed as described in the main manuscript. The predictors’ measurement model relied on an Exploratory Structural Equation Model (ESEM) specification (Morin et al., 2013, 2020). This decision was predicated on the mounting statistical evidence supporting the value of incorporating the free estimation of cross-loadings via an ESEM solution for any multidimensional measures in order to achieve a more accurate (i.e., not inflated) estimate of factor correlations (e.g., Asparouhov et al., 2015). In the present study, all multi-item predictors came from the same multidimensional measure, and avoiding multicollinearity appeared to be particularly important in the assessment of the relative, and unique, effects of these various work characteristics. This model also included an orthogonal method factor to control for the methodological artefact related to the negative wording of six of the items (Marsh et al., 2010). This model resulted in satisfactory level of fit to the data (*χ^2^* = 355.621, *df* = 110, *p* ≤ .01; CFI = .950; TLI = .914; RMSEA = .047; RMSEA CI = .041 to .052). The parameter estimates from this model are reported in Table S4 of these supplements, and reveal well-defined (ω = .709 for workload, .714 for control, .750 for community, .706 for fairness), and relatively independent (*|r|* = .320 to .517), factors with few noteworthy cross-loadings (only one cross loading was higher than .400, and one more was higher than .300).

For the outcomes, given that all measures came from distinct instruments and that factor correlations among outcomes are unlikely to impact the key relations estimated in the present study, we relied on a more classical confirmatory factor analysis (CFA) representation of participants’ levels of job satisfaction, turnover intentions, psychological distress, and vitality. In this model, each item was only allowed to load on the factor it was assumed to measure, with no cross-loadings allowed, and the factors were allowed to correlate. This model resulted in acceptable level of fit to the data (*χ^2^* = 811.372, *df* = 164, *p* ≤ .01; CFI = .923; TLI = .910; RMSEA = .062; RMSEA CI = .058 to .066). The parameter estimates from this model are reported in Table S5 of these supplements, and reveal well-defined (ω = .864 for job satisfaction, .755 for turnover intentions, .865 for psychological distress, and .892 for vitality), and relatively independent (*|r|* = .195 to .601), factors.

**References**

Asparouhov, T., Muthén, B., & Morin, A.J.S. (2015). Bayesian structural equation modeling with cross-loadings and residual covariances. *Journal of Management, 41*, 1561-1577.

Chen, F.F. (2007). Sensitivity of goodness of fit indexes to lack of measurement. *Structural Equation Modeling, 14*, 464-504.

Cheung, G.W., & Rensvold, R.B. (2002). Evaluating goodness-of fit indexes for testing measurement invariance. *Structural Equation Modeling, 9*, 233-255.

Garn, A.C., Morin, A.J.S., & Lonsdale, C. (2018). Basic psychological need satisfaction toward learning: A longitudinal test of mediation using bifactor exploratory structural equation modeling. *Journal of Educational* Psychology, 111, 354–372.

Gillet, N., Morin, A.J.S., Choisay, F. & Fouquereau, E. (2019). A person-centered representation of basic need satisfaction balance at work. *Journal of Personnel Psychology, 18*, *113-128*.

Gillet, N., Morin, A.J.S., Huyghebaert-Zouaghi, T., Alibran, E., Barrault, S., & Vanhove-Meriaux, C. (2020). Students’ Need Satisfaction Profiles: Similarity and Change over the Course of a University Semester. *Applied Psychology: An International* *Review*. Early view. doi: 10.1111/apps.12227

Hu, L.-T., & Bentler, P.M. (1999). Cutoff criteria for fit indexes in covariance structure analysis: Conventional criteria versus new alternatives. *Structural Equation Modeling, 6,* 1-55.

Marsh, H.W. (1996). Positive and negative global self-esteem: A substantively meaningful distinction or artifactors?. *Journal of Personality and Social Psychology, 70*, 810-819.

Marsh, H.W. (2007). Application of confirmatory factor analysis and structural equation modeling in sport/exercise psychology. In G. Tenenbaum & R.C. Eklund (Eds.), *Handbook of sport psychology* (3^rd^ ed., pp. 774-798). New York, NY: Wiley.

Marsh, H.W., Abduljabbar, A.S., Abu-Hilal, M., Morin, A.J.S., Abdelfattah, F., Leung, K.C., Xu, M.K., Nagengast, B., & Parker, P. (2013). Factorial, convergent, and discriminant validity of TIMSS math and science motivation measures: A comparison of Arab and Anglo-Saxon Countries. *Journal of Educational Psychology, 105,* 108-128.

Marsh, H.W., Scalas, L.F., & Nagengast, B. (2010). Longitudinal tests of competing factor structures for the Rosenberg self-esteem scale: Traits, ephemeral artifacts, and stable response styles. *Psychological Assessment, 22,* 366-381.

Marsh, H.W., Hau, K., & Grayson, D. (2005). Goodness of fit in structural equation models. In A. Maydeu-Olivares & J.J. McArdle (Eds.), *Contemporary psychometrics* (pp. 275-340). Mahwah, NJ: Erlbaum.

McDonald, R.P. (1970). Theoretical foundations of principal factor analysis, canonical factor analysis, and alpha factor analysis. *British Journal of Mathematical & Statistical Psychology, 23*, 1-21.

Morin, A.J.S., Marsh, H.W., & Nagengast, B. (2013). Exploratory Structural Equation Modeling. In G.R. Hancock & R.O. Mueller (Eds.), Structural Equation Modeling: A Second Course, 2nd Edition (pp. 395-436). Greewich, Connecticut: IAP.

Morin, A. J. S., Myers, N. D., & Lee, S. (2020). Modern factor analytic techniques: Bifactor models, exploratory structural equation modeling (ESEM) and bifactor-ESEM. In G. Tenenbaum & R.C. Eklund (Eds.), *Handbook of sport psychology* (4^th^ ed; pp. 1044-1073). London, UK: Wiley.

Muthén, L.K., & Muthén, B. (2019). *Mplus user’s guide.* Los Angeles: Muthén & Muthén.

Perreira, T.A., **Morin, A.J.S.,** Hebert, M., Gillet, N., Houle, S.A., & Berta, W. (2018). The short form of the Workplace Affective Commitment Multidimensional Questionnaire (WACMQ-S): A bifactor-ESEM approach among healthcare professionals. Journal of Vocational Behavior, 106, 62-83

Tóth-Király, I., Morin, A.J.S., Bőthe, B., Orosz, G., & Rigó, A. (2018). Investigating the multidimensionality of need fulfillment: A bifactor exploratory structural equation modeling representation. *Structural Equation Modeling, 25,* 267-286.

*Table S1.*

Goodness-of-Fit Statistics for the Measurement Models

| Model | *χ*² (*df*) | CFI | TLI | RMSEA | 90% CI |
| --- | --- | --- | --- | --- | --- |
| *Measurement Models: Need Satisfaction* |  |  |  |  |  |
| A priori CFA (6 domain-specific factors) | 330.108 (111)* | .940 | .918 | .044 | [.038; .049] |
| Bifactor CFA-1 (1 domain-general G-factor, 6 domain-specific S-factors) | 262.319 (99)* | .956 | .931 | .040 | [.034; .046] |
| Bifactor CFA-2 (2 domain-specific G-factors, 6 domain-specific S-factors) | 235.525 (98)* | .963 | .942 | .037 | [.031; .043] |
| Bifactor CFA-3 (1 domain-general G-factor, 3 domain-general S-factors) | 1108.824 (108)* | .728 | .614 | .095 | [.090; .100] |
| Bifactor CFA-4 (2 domain-specific G-factors, 3 domain-general S-factors) | 332.362 (107)* | .939 | .912 | .045 | [.040; .051] |
| *Tests of Differential Item Functioning: Need Satisfaction* | |  |  |  |  |
| Null | 764.445 (143)* | .853 | .806 | .065 | [.061; .070] |
| Saturated | 307.084 (107)* | .953 | .917 | .043 | [.037; .048] |
| Invariant | 363.196 (133)* | .946 | .923 | .041 | [.036; .046] |

*Note:* * *p*< .01; *χ*²: Scaled chi-square test of exact fit; *df*: Degrees of freedom; CFI: Comparative fit index; TLI: Tucker–Lewis index; RMSEA: Root mean square error of approximation; 90% CI: 90% confidence interval.

*Table S2.*

Standardized Factor Loadings (λ) and Uniquenesses (δ) from the A Priori CFA Solution (Six Domain-Specific Factors)

| Item | Work  Autonomy λ | Work  Competence λ | | Work  Relatedness λ | | Work  δ | | Personal  Autonomy λ | | Personal  Competence λ | | Personal  Relatedness λ | | Personal  δ | |
| --- | --- | --- | --- | --- | --- | --- | --- | --- | --- | --- | --- | --- | --- | --- | --- |
| Autonomy |  |  | |  | |  | |  | |  | |  | |  | |
| Item 1 | .513 |  | |  | | .737 | | .392 | |  | |  | | .846 | |
| Item 2 | .690 |  | |  | | .524 | | .617 | |  | |  | | .619 | |
| Item 3 | .760 |  | |  | | .422 | | .786 | |  | |  | | .382 | |
| ω | .696 |  | |  | |  | | .636 | |  | |  | |  | |
| Competence |  |  | |  | |  | |  | |  | |  | |  | |
| Item 1 |  | .166 | |  | | .972 | |  | | .206 | |  | | .958 | |
| Item 2 |  | .490 | |  | | .760 | |  | | .587 | |  | | .655 | |
| Item 3 |  | .699 | |  | | .511 | |  | | .711 | |  | | .494 | |
| ω |  | .450 | |  | |  | |  | | .518 | |  | |  | |
| Relatedness |  |  | |  | |  | |  | |  | |  | |  | |
| Item 1 |  |  | | .614 | | .624 | |  | |  | | .633 | | .600 | |
| Item 2 |  |  | | .323 | | .896 | |  | |  | | .394 | | .845 | |
| Item 3 |  |  | | .791 | | .375 | |  | |  | | .743 | | .447 | |
| ω |  |  | | .612 | |  | |  | |  | | .623 | |  | |
|  | Work Autonomy | | Work Competence | | Work Relatedness | | Personal Autonomy | | Personal Competence | | Personal Relatedness | |  | |  |
| Work Autonomy | - |  | |  | |  | |  | |  | |  | |  | |
| Work Competence | .961* | - | |  | |  | |  | |  | |  | |  | |
| Work Relatedness | .817* | .896* | | - | |  | |  | |  | |  | |  | |
| Personal Autonomy | .175* | .154* | | .198* | | - | |  | |  | |  | |  | |
| Personal Competence | .102* | .168* | | .240* | | .889* | | - | |  | |  | |  | |
| Personal Relatedness | .115* | .098 | | .341* | | .872* | | .834* | | - | |  | |  | |

*Note*. λ: Factor loading; δ: Item uniqueness; ω: Omega coefficient of model-based composite reliability; non-significant parameters (*p* ≥ .05) are marked in italics.

*Table S3.*

*Standardized Factor Loadings (λ) and Uniquenesses (δ) from the Bifactor CFA-Four solution (Two Domain-Specific G-Factors, Three Domain-General S-Factors)*

| Items | Work  G-Factor λ | Personal  G-Factor λ | Autonomy  S-Factor λ | Competence  S-Factor λ | Relatedness  S-Factor λ | δ |
| --- | --- | --- | --- | --- | --- | --- |
| Work Autonomy Items |  |  |  |  |  |  |
| Item 1 | .498 |  | .458 |  |  | .542 |
| Item 2 | .676 |  | *.022* |  |  | .543 |
| Item 3 | .734 |  | *.048* |  |  | .458 |
| Personal Autonomy Items |  |  |  |  |  |  |
| Item 1 |  | .377 | .926 |  |  | *.001* |
| Item 2 |  | .602 | .091 |  |  | .630 |
| Item 3 |  | .782 | *-.013* |  |  | .389 |
| ω |  |  | .486 |  |  |  |
| Work Competence Items |  |  |  |  |  |  |
| Item 1 | .167 |  |  | *.219* |  | .924 |
| Item 2 | .487 |  |  | *.201* |  | .723 |
| Item 3 | .702 |  |  | *-.013* |  | .507 |
| Personal Competence Items |  |  |  |  |  |  |
| Item 1 |  | .186 |  | *.050* |  | .963 |
| Item 2 |  | .526 |  | .446 |  | *.524* |
| Item 3 |  | .650 |  | *.165* |  | .551 |
| ω |  |  |  | .222 |  |  |
| Work Relatedness Items |  |  |  |  |  |  |
| Item 1 | .514 |  |  |  | .478 | .507 |
| Item 2 | .317 |  |  |  | *.106* | .888 |
| Item 3 | .680 |  |  |  | .278 | .460 |
| Personal Relatedness Items |  |  |  |  |  |  |
| Item 1 |  | .551 |  |  | .434 | .508 |
| Item 2 |  | .324 |  |  | .207 | .852 |
| Item 3 |  | .666 |  |  | .183 | .523 |
| ω | .804 | .815 |  |  | .432 |  |

*Note*. G = Global factor estimated as part of a bifactor model; S = Specific factor estimated as part of a bifactor model; λ: Factor loading; δ: Item uniqueness; ω: Omega coefficient of model-based composite reliability; non-significant parameters (*p* ≥ .05) are marked in italics

*Table S4.*

Standardized Factor Loadings (λ) and Uniquenesses (δ) for the Predictors’ Measurement Model

|  | Factor 1 | Factor 2 | Factor 3 | Factor 4 |  |
| --- | --- | --- | --- | --- | --- |
| Items | λ | λ | λ | λ | δ |
| Workload |  |  |  |  |  |
| Item 1 | **.731** | *.068* | *.033* | -.100 | .459 |
| Item 2 | **.441** | *.044* | .105 | -.111 | .801 |
| Item 3 | **.581** | *-.019* | *-.025* | *-.026* | .632 |
| Item 4 | **.613** | -.178 | *.038* | *-.044* | .510 |
| Item 5 | **.421** | .226 | -.106 | .196 | .781 |
| ω | .709 |  |  |  |  |
| Control |  |  |  |  |  |
| Item 1 | *-.031* | **.583** | *.055* | -.251 | .717 |
| Item 2 | *.035* | **.343** | *-.016* | .427 | .575 |
| Item 3 | *-.020* | **.798** | *.027* | -.160 | .442 |
| Item 4 | .058 | **.634** | *-.007* | .171 | .490 |
| ω |  | .714 |  |  |  |
| Community |  |  |  |  |  |
| Item 1 | *-.067* | .092 | **.488** | *.087* | .626 |
| Item 2 | .083 | .145 | **.395** | .242 | .631 |
| Item 3 | *-.035* | *-.067* | **.817** | *.000* | .364 |
| Item 4 | .073 | *-.035* | **.794** | *-.008* | .430 |
| Item 5 | *.001* | *.006* | **.438** | *-.063* | .822 |
| ω |  |  | .750 |  |  |
| Fairness |  |  |  |  |  |
| Item 1 | -.192 | *.087* | .170 | **.393** | .587 |
| Item 2 | *.022* | *.000* | *.054* | **.541** | .681 |
| Item 3 | *-.029* | .370 | *-.034* | **.431** | .521 |
| Item 4 | *-.050* | *.103* | .142 | **.565** | .481 |
| Item 5 | -.150 | .139 | .111 | **.334** | .555 |
| Item 6 | *-.043* | .110 | *.053* | **.523** | *.402* |
| ω |  |  |  | .706 |  |
| *Correlations* | 1. | 2. | 3. | 4. |  |
| 1. Workload |  |  |  |  |  |
| 2. Control | -.332 |  |  |  |  |
| 3. Community | -.320 | .483 |  |  |  |
| 4. Fairness | -.325 | .517 | .405 |  |  |

*Note*. λ: Factor loading; δ: Item uniqueness; ω: Omega coefficient of model-based composite reliability; target ESEM factor loadings are indicated in bold; non-significant parameters (*p* ≥ .05) are marked in italics.

*Table S5.*

Standardized Factor Loadings (λ) and Uniquenesses (δ) for the Outcomes’ Measurement Model

|  | Satisfaction | Turnover Intentions | Psychological Distress | Vitality |  |
| --- | --- | --- | --- | --- | --- |
| Items | λ | λ | λ | λ | δ |
| Job Satisfaction |  |  |  |  |  |
| Item 1 | .704 |  |  |  | .505 |
| Item 2 | .740 |  |  |  | .453 |
| Item 3 | .785 |  |  |  | .384 |
| Item 4 | .779 |  |  |  | .393 |
| Item 5 | .733 |  |  |  | .463 |
| ω | .864 |  |  |  |  |
| Turnover Intentions |  |  |  |  |  |
| Item 1 |  | .792 |  |  | .373 |
| Item 2 |  | .888 |  |  | .211 |
| Item 3 |  | .411 |  |  | .831 |
| ω |  | .755 |  |  |  |
| Psychological Distress |  |  |  |  |  |
| Item 1 |  |  | .657 |  | .568 |
| Item 2 |  |  | .830 |  | .311 |
| Item 3 |  |  | .617 |  | .620 |
| Item 4 |  |  | .795 |  | .368 |
| Item 5 |  |  | .712 |  | .494 |
| Item 6 |  |  | .685 |  | .531 |
| ω |  |  | .865 |  |  |
| Vitality |  |  |  |  |  |
| Item 1 |  |  |  | .815 | .335 |
| Item 2 |  |  |  | .638 | .593 |
| Item 3 |  |  |  | .775 | .399 |
| Item 4 |  |  |  | .698 | .513 |
| Item 5 |  |  |  | .790 | .376 |
| Item 6 |  |  |  | .840 | .294 |
| ω |  |  |  | .892 |  |
| *Correlations* | 1. | 2. | 3. | 4. |  |
| 1. Job Satisfaction |  |  |  |  |  |
| 2. Turnover Intentions | -.601 |  |  |  |  |
| 3. Psych. Distress | -.503 | .417 |  |  |  |
| 4. Vitality | .420 | -.195 | -.385 |  |  |

*Note*. λ: Factor loading; δ: Item uniqueness; ω: Omega coefficient of model-based composite reliability; Psych. = Psychological.

*Table S6.*

Correlations between all Variables Used in the Present Study

| Variable | 1. | 2. | 3. | 4. | 5. | 6. | 7. | 8. | 9. | 10. | 11. | 12. | 13. | 14. |
| --- | --- | --- | --- | --- | --- | --- | --- | --- | --- | --- | --- | --- | --- | --- |
| 1. Sex | - |  |  |  |  |  |  |  |  |  |  |  |  |  |
| 2. Age | -.138** |  |  |  |  |  |  |  |  |  |  |  |  |  |
| 3. Org. tenure | -.077* | .349** |  |  |  |  |  |  |  |  |  |  |  |  |
| 4. Education | .100** | -.100** | -.023 |  |  |  |  |  |  |  |  |  |  |  |
| 5. Rel. Status | -.058 | .170** | .022 | -.032 |  |  |  |  |  |  |  |  |  |  |
| 6. #Children | -.092** | .269** | .042 | -.017 | .320** |  |  |  |  |  |  |  |  |  |
| 7. Autonomy NS^1^ | -.023 | .024 | -.016 | -.076* | -.020 | -.067* | - |  |  |  |  |  |  |  |
| 8. Competence NS^1^ | .037 | .035 | .006 | .039 | .037 | .011 | -.012 | - |  |  |  |  |  |  |
| 9. Relatedness NS^1^ | .200** | -.070* | .046 | .107** | -.026 | -.051 | -.039 | -.032 | - |  |  |  |  |  |
| 10. Global NS: Work^1^ | .024 | .054 | .020 | .059 | .052 | .080* | .080** | .041 | .196** | - |  |  |  |  |
| 11. Global NS: Personal^1^ | .162** | -.058 | .004 | .040 | .043 | -.096** | .091** | .166** | .241** | .144** | - |  |  |  |
| 12. Workload^1^ | .008 | -.025 | -.010 | -.013 | .041 | -.035 | -.306** | -.058 | -.087** | -.433** | -.045 | - |  |  |
| 13. Control^1^ | -.041 | .053 | -.017 | .096** | .060 | .119** | .124** | .008 | .067* | .708** | .038 | -.415** | - |  |
| 14. Community^1^ | -.056 | .012 | .055 | .075* | .014 | .056 | .088** | .002 | .280** | .589** | .101** | -.391** | .576** | - |
| 15. Fairness | .035 | .044 | -.035 | -.027 | .038 | .036 | .158** | -.086** | .168** | .631** | .080* | -.443** | .668** | .520** |
| 16. Job satisfaction^1^ | .002 | .042 | -.012 | .031 | .070* | .064* | .186** | .020 | .168** | .724** | .104** | -.504** | .642** | .529** |
| 17. Turnover intentions^1^ | .047 | -.164** | -.041 | .087** | -.068* | -.102** | -.131** | -.034 | -.006 | -.498** | .065* | .381** | -.424** | -.370** |
| 18. Psychological distress^1^ | .072* | -.070* | -.011 | -.073* | -.070* | -.004 | -.349** | -.057 | -.109** | -.446** | -.188** | .447** | -.385** | -.355** |
| 19. Vitality^1^ | .045 | .072* | -.013 | .050 | .024 | .103** | .136** | .175** | .151** | .411** | .266** | -.193** | .300** | .235** |
| 20. Absences (Occurrence) | .144** | -.076* | -.021 | -.098** | -.034 | -.033 | -.118** | .038 | -.008 | -.154** | .011 | .121** | -.214** | -.150** |
| 21. Absences (Days missed) | -.001 | .061* | -.115** | -.100** | .016 | .031 | -.014 | .075* | -.016 | -.145** | -.014 | .053 | -.164** | -.127** |
| 22. Work injury (Incidents) | -.047 | -.029 | -.073* | -.064* | -.001 | -.018 | -.015 | .028 | -.089** | -.115** | -.090** | .097** | -.125** | -.069* |
| 23. Work injury (Days Missed) | -.012 | -.036 | -.066* | -.023 | -.022 | -.003 | -.008 | .032 | -.073* | -.045 | -.084** | .063* | -.074* | -.032 |
| 24. Absenteeism (Days) | .001 | .035 | -.113** | -.073* | .007 | -.009 | -.029 | .069* | .004 | -.116** | -.012 | .049 | -.132** | -.126** |
| 25. Presenteeism | -.068* | .018 | -.076* | -.117** | .019 | .035 | -.027 | .014 | -.072* | -.106** | .003 | .067* | -.123** | -.108** |
| 26. Performance | -.004 | -.073* | .009 | -.054 | -.078* | -.062* | -.145** | -.100** | -.061* | -.435** | -.088** | .282** | -.378** | -.270** |

*Note*. * *p* < .05; ** *p* < .01; ^1^: indicators are estimated from factor scores with a standard deviation of 1 and a mean of 0; NS: Need satisfaction.

*Table S6 (Continued).*

Correlations between all Variables Used in the Present Study

| Variable | 15. | 16. | 17. | 18. | 19. | 20. | 21. | 22. | 23. | 24. | 25. | 26. |
| --- | --- | --- | --- | --- | --- | --- | --- | --- | --- | --- | --- | --- |
| 15. Fairness | - |  |  |  |  |  |  |  |  |  |  |  |
| 16. Job satisfaction^1^ | .658** | - |  |  |  |  |  |  |  |  |  |  |
| 17. Turnover intentions^1^ | -.447** | -.673** | - |  |  |  |  |  |  |  |  |  |
| 18. Psychological distress^1^ | -.382** | -.563** | .472** | - |  |  |  |  |  |  |  |  |
| 19. Vitality^1^ | .268** | .465** | -.223** | -.426** | - |  |  |  |  |  |  |  |
| 20. Absences (Occurrence) | -.159** | -.193** | .181** | .214** | -.110** | - |  |  |  |  |  |  |
| 21. Absences (Days missed) | -.113** | -.148** | .046 | .154** | -.104** | .439** | - |  |  |  |  |  |
| 22. Work injury (Incidents) | -.104** | -.100** | .062* | .083** | -.055 | .081** | .023 | - |  |  |  |  |
| 23. Work injury (Days Missed) | -.026 | -.042 | .025 | .027 | -.010 | .023 | -.002 | .670** | - |  |  |  |
| 24. Absenteeism (Days) | -.083** | -.131** | .052 | .162** | -.099** | .265** | .768** | .153** | .271** | - |  |  |
| 25. Presenteeism | -.110** | -.099** | .031 | .107** | -096** | .058 | .102** | .084** | .057 | .105** | - |  |
| 26. Performance | -.315** | -.453** | .392** | .355** | -.315** | .107** | .056 | -.017 | -.031 | .034 | .050 | - |

*Note*. * *p* < .05; ** *p* < .01; ^1^: indicators are estimated from factor scores with a standard deviation of 1 and a mean of 0; NS: Need satisfaction.

*Table S7.*

Classification Accuracy: Classification Probability for Most Likely Profile Membership (Column) as a Function of the Profile Membership (Row).

|  | Profile 1 | Profile 2 | Profile 3 | Profile 4 | Profile 5 |
| --- | --- | --- | --- | --- | --- |
| Profile 1 | .797 | .077 | .055 | .065 | .006 |
| Profile 2 | .026 | .820 | .023 | .050 | .080 |
| Profile 3 | .035 | .074 | .810 | .058 | .023 |
| Profile 4 | .057 | .152 | .140 | .639 | .013 |
| Profile 5 | .001 | .064 | .019 | .004 | .912 |

Note. Profile 1: *Globally Unsatisfied*; Profile 2: *Globally Satisfied in Personal Life with High Autonomy*; Profile 3: *Globally Satisfied in Personal Life with Low Autonomy*; Profile 4: *Globally Satisfied at Work with High Relatedness*; and Profile 5: *Globally Satisfied*.

*Table S8.*

Detailed Results from the Final Latent Profile Solution

|  | Profile 1 | Profile 2 | Profile 3 | Profile 4 | Profile 5 |
| --- | --- | --- | --- | --- | --- |
| *Means* | Mean [CI] | Mean [CI] | Mean [CI] | Mean [CI] | Mean [CI] |
| Autonomy (domain-general, S-factor) | -.152 [-.364; .060] | .639 [.517; .761] | -.969 [-1.153; -.785] | -.281 [-.563; .001] | .601 [.499; .703] |
| Competence (domain-general, S-factor) | -.161 [-.296; -.026] | .001 [-.077; .079] | .214 [.147; .281] | -.243 [-.410; -.076] | .249 [.163; .335] |
| Relatedness (domain-general, S-factor) | -.461 [-.639; -.283] | -.018 [-.200; .164] | .222 [.042; .402] | .311 [.142; .480] | .187 [.073; .301] |
| Global (work domain, G-factor) | -.420 [-.589; -.251] | .035 [-.157; .227] | -.096 [-.314; .122] | .561 [.287; .835] | .150 [-.066; .366] |
| Global (personal domain, G-factor) | -1.028 [-1.238; -.818] | .323 [.180; .466] | .313 [.182; .444] | -.145 [-.463; .173] | .884 [.835; .933] |
| *Variances* | Variance [CI] | Variance [CI] | Variance [CI] | Variance [CI] | Variance [CI] |
| Autonomy (domain-general, S-factor) | 1.665 [1.408; 1.922] | .169 [.118; .220] | .531 [.215; .847] | .848 [.472; 1.224] | .176 [.113; .239] |
| Competence (domain-general, S-factor) | .577 [.459; .695] | .177 [.122; .232] | .071 [.044; .098] | .350 [.148; .552] | .063 [.038; .088] |
| Relatedness (domain-general, S-factor) | .720 [.593; .847] | .358 [.242; .474] | .285 [.150; .420] | .161 [.047; .275] | .224 [.138; .310] |
| Global (work domain, G-factor) | .627 [.505; .749] | .858 [.719; .997] | .783 [.620; .946] | .176 [.002; .350] | 1.162 [.935; 1.389] |
| Global (personal domain, G-factor) | 1.121 [.894; 1.348] | .123 [.080; .166] | .160 [.109; .211] | .271 [.214; .328] | .010 [.002; .018] |

*Note*. CI = 95% confidence interval; the profile indicators are estimated from factor scores with a standard deviation of 1 and a mean of 0; Profile 1: *Globally Unsatisfied*; Profile 2: *Globally Satisfied in Personal Life with High Autonomy*; Profile 3: *Globally Satisfied in Personal Life with Low Autonomy*; Profile 4: *Globally Satisfied at Work with High Relatedness*; and Profile 5: *Globally Satisfied*.

1. Although allowing S-factors to correlate across domains may seem to violate the orthogonality assumptions of bifactor models (Morin et al., 2020), these assumptions only hold for a specific set of *bifactor* factors. Here, two sets of *bifactor* factors are incorporated into the same model. However, re-estimating the first and second bifactor alternatives to assume the complete orthogonality of the S-factors did not change our conclusions regarding the optimal solution (results available upon request). [↑](#footnote-ref-1)
